# Neuronally Derived Extracellular Vesicle α-Synuclein as a Serum Biomarker for Individuals at Risk of Developing Parkinson Disease

**DOI:** 10.1001/jamaneurol.2023.4398

**Published:** 2023-12-04

**Authors:** Shijun Yan, Cheng Jiang, Annette Janzen, Thomas R. Barber, Aline Seger, Michael Sommerauer, Jason J. Davis, Kenneth Marek, Michele T. Hu, Wolfgang H. Oertel, George K. Tofaris

**Affiliations:** 1 Nuffield Department of Clinical Neurosciences, University of Oxford, Oxford, United Kingdom; 2 Kavli Institute for Nanoscience Discovery, University of Oxford, Oxford, United Kingdom; 3 Department of Neurology, Philipps-University Marburg, Marburg, Germany; 4 Oxford Parkinson’s Disease Centre, University of Oxford, Oxford, United Kingdom; 5 Department of Neurology, University Hospital Cologne, Faculty of Medicine, University of Cologne, Köln, Germany; 6 Institute of Neuroscience and Medicine (INM-3), Forschungszentrum Jülich, Jülich, Germany; 7 Department of Chemistry, University of Oxford, Oxford, United Kingdom; 8 Institute for Neurodegenerative Disorders, New Haven, Connecticut

## Abstract

**IMPORTANCE:**

Nonmotor symptoms of Parkinson disease (PD) often predate the movement disorder by decades. Currently, there is no blood biomarker to define this prodromal phase.

**OBJECTIVE:**

To investigate whether α-synuclein in neuronally derived serum-extracellular vesicles identifies individuals at risk of developing PD and related dementia.

**DESIGN, SETTING, and PARTICIPANTS:**

This retrospective, cross-sectional multicenter study of serum samples included the Oxford Discovery, Marburg, Cologne, and Parkinson’s Progression Markers Initiative cohorts. Participants were recruited from July 2013 through August 2023 and samples were analyzed from April 2022 through September 2023. The derivation group (n = 170) included participants with isolated rapid eye movement sleep behavior disorder (iRBD) and controls. Two validation groups were used: the first (n = 122) included participants with iRBD and controls and the second (n = 263) included nonmanifest *GBA1^N409S^* gene carriers, participants with iRBD or hyposmia, and available dopamine transporter single-photon emission computed tomography, healthy controls, and patients with sporadic PD. Overall the study included 199 participants with iRBD, 20 hyposmic participants with available dopamine transporter single-photon emission computed tomography, 146 nonmanifest *GBA1^N409S^* gene carriers, 21 *GBA1^N409S^* gene carrier patients with PD, 50 patients with sporadic PD, and 140 healthy controls. In the derivation group and validation group 1, participants with polysomnographically confirmed iRBD were included. In the validation group 2, at-risk participants with available Movement Disorder Society prodromal markers and serum samples were included. Among 580 potential participants, 4 were excluded due to alternative diagnoses.

**EXPOSURES:**

Clinical assessments, imaging, and serum collection.

**MAIN OUTCOME AND MEASURES:**

L1CAM-positive extracellular vesicles (L1EV) were immunocaptured from serum. α-Synuclein and syntenin-1 were measured by electrochemiluminescence. Area under the receiver operating characteristic (ROC) curve (AUC) with 95% CIs evaluated biomarker performance. Probable prodromal PD was determined using the updated Movement Disorder Society research criteria. Multiple linear regression models assessed the association between L1EV α-synuclein and prodromal markers.

**RESULTS:**

Among 576 participants included, the mean (SD) age was 64.30 (8.27) years, 394 were male (68.4%), and 182 were female (31.6%). A derived threshold of serum L1EV α-synuclein distinguished participants with iRBD from controls (AUC = 0.91; 95% CI, 0.86-0.96) and those with more than 80% probability of having prodromal PD from participants with less than 5% probability (AUC = 0.80; 95% CI, 0.71-0.89). Subgroup analyses revealed that specific combinations of prodromal markers were associated with increased L1EV α-synuclein levels. Across all cohorts, L1EV α-synuclein differentiated participants with more than 80% probability of having prodromal PD from current and historic healthy control populations (AUC = 0.90; 95% CI, 0.87-0.93), irrespective of initial diagnosis. L1EV α-synuclein was increased in at-risk participants with a positive cerebrospinal fluid seed amplification assay and was above the identified threshold in 80% of cases (n = 40) that phenoconverted to PD or related dementia.

**CONCLUSIONS AND RELEVANCE:**

L1EV α-synuclein in combination with prodromal markers should be considered in the stratification of those at high risk of developing PD and related Lewy body diseases.

## Key Points

**Question:**

Is neuronally derived extracellular vesicle-associated α-synuclein in serum a biomarker for Parkinson disease pathology in at-risk individuals?

**Findings:**

In this cross-sectional study that included 576 participants, serum L1CAM-positive extracellular vesicle (L1EV)-associated α-synuclein differentiated at-risk participants with more than 80% probability of having prodromal Parkinson disease from controls and correctly identified 80% of those who phenoconverted to Parkinson disease and related dementia.

**Meaning:**

Measurement of L1EV α-synuclein levels in serum could be used as a screening test of Parkinson pathology in at-risk individuals.

## Teaser

This study investigate whether α-synuclein in neuronally derived serum-extracellular vesicles identifies individuals at risk of developing Parkinson disease and related dementia.

## Introduction

Parkinson disease (PD) is the most common movement disorder, characterized by a long prodromal phase, which starts several years before clinical presentation with the classic motor symptoms. A number of nonmotor symptoms, such as rapid eye movement sleep behavior disorder (RBD), olfactory loss, or autonomic dysfunction can manifest during the prodromal phase and are associated with higher risk of phenoconversion to PD.^1^ Isolated RBD (iRBD) is the strongest predictor of α-synucleinopathy with more than 80% of participants converting to PD, dementia with Lewy bodies (DLB), or multiple system atrophy (MSA) within 12 years.^2^ Participants with heterozygous mutations in the *glucocerebrosidase (GBA1*) gene are another recognized at-risk group with an approximately 6% to 10% risk of developing PD by age 70 years,^3^ which is much lower than the respective risk for iRBD. Identification of those individuals with the highest risk of phenoconversion is crucial for the assessment of future disease-modifying therapies within reasonable timescales. Because symptoms broadly correlate with the evolution of α-synuclein pathology, accurate measurements of neuronally derived α-synuclein in at-risk individuals, preferably from an easily accessible biosample, such as blood, could aid the identification of those likely to convert to PD.

Extracellular vesicles (EVs) derived from tissues affected by disease have emerged as a rich source of biomarkers.^4,5^ Furthermore, immunocapture of presumed neuronally derived circulating EVs using anti-L1CAM (L1EVs) has been applied to a number of neurologic diseases as a proxy biomarker of brain pathology.^6^ Despite this extensive literature, the precise relevance of L1EVs in PD diagnostics remains unresolved. We previously showed in multiple cohorts (more than 800 participants) that α-synuclein is elevated in L1EVs isolated from patients with PD, PD dementia (PDD), or neuropathologically confirmed DLB and in a single cohort of participants with polysomnographically confirmed iRBD but not in other parkinsonian sydromes.^7,8^ This finding is consistent with the view that L1EV-associated α-synuclein levels are higher in patients with PD based on several studies from our and other laboratories.^7,8,9,10,11,12^ We previously proposed that the most valuable application of L1EV-associated α-synuclein measurements is in the prediction or differentiation of PD from phenotypically similar parkinsonian symptoms, including MSA.^7,8^ The goals of this study were to determine whether serum L1EV-associated α-synuclein identifies participants in the prodromal phase of α-synucleinopathy, especially those participants at high risk of developing PD, establish assay sensitivity and specificity using a large number of samples across cohorts, and investigate the performance of this blood-based test in relation to dopaminergic neurodegeneration, as assessed by dopamine transporter single-photon emission computed tomography (DaT SPECT), pathology as assessed by α-synuclein seed amplification assay (SAA) in cerebrospinal fluid (CSF) and phenoconversion.

## Methods

### Study Design

This is a retrospective cross-sectional study of 4 cohorts that followed the Strengthening the Reporting of Observational Studies in Epidemiology (STROBE) reporting guidelines. Written consent was obtained from all participants and the protocols followed the principles of the Declaration of Helsinki and were approved by the respective ethics committees for each site. The study profile for the derivation and validation groups is shown in eFigure 1 in Supplement 1. The biomarker was first tested across homogeneous single-center iRBD cohorts with Oxford Discovery as the derivation group and the identified cutoff (derived threshold) was assessed in the combined Marburg+Cologne cohort as the first validation group. Inclusion criteria for iRBD were confirmation by video-assisted polysomnography and available serum samples. Participants were excluded if a secondary cause for RBD was present. Oxford iRBD participants (n = 97) were recruited from the Discovery cohort of the Oxford Parkinson Disease Centre.^13,14^ Marburg iRBD participants (n = 51) were recruited nationwide from the German RBD registry project and annually followed up with for at least 4 years at Marburg by the same neurologist (A.J.).^15^ iRBD Participants from Cologne (n = 24) were identified from the general population using a structured screening process after written informed consent was given.^16^ Healthy controls from Oxford (n = 73), Marburg (n = 29), and Cologne (n = 18) without significant comorbidities or relevant family history were included as reference groups. The threshold derived from the Oxford Discovery cohort was further assessed in the second validation group consisting of participants with variable risk from the Parkinson’s Progression Markers Initiative (PPMI) cohort. Inclusion criteria for PPMI were available serum samples and assessments for prodromal PD. These participants were recruited across sites in the US, Europe, or Israel^17^and included nonmanifest *GBA1^N409S^* gene carriers (n = 146), participants with hyposmia (n = 20), or polysomnographically confirmed iRBD (n = 27). Healthy controls without relevant comorbidities (n = 20), *GBA1^N409S^* PD (n = 21) and sporadic PD (n = 50) were included as reference groups. Subgroup analyses within each prodromal condition were performed to determine the performance of the test when more than 1 prodromal marker was present. The probability of having prodromal PD was estimated across cohorts as detailed in eMethods 1 in Supplement 1 and samples were analyzed blinded to the phenotype. Serum samples were matched to the clinical assessments. L1EVs were isolated and α-synuclein and syntenin-1 were measured as detailed in eMethods 2 in Supplement 1 without noticeable batch effects as shown in eFigure 2 in Supplement 1. Where such results were available, we also assessed the association between serum L1EV α-synuclein levels and CSF total α-synuclein levels or CSF α-synuclein SAA or phenoconversion.

### Statistical Analysis

For multiple comparisons of L1EV α-synuclein measurements, we performed nonparametric statistical testing as the data were not normally distributed: Kruskal-Wallis 1-way analysis of variance with the Dunn test for post hoc comparison between individual pairings when there were 3 or more independent groups and Mann-Whitney *U* test when there were only 2 independent groups. Data from different groups were analyzed using receiver operating characteristic (ROC) with 95% CIs (2.5%-97.5%). The optimum cutoff point (derived threshold) was determined by the Youden index, ie, the value associated with the maximal value of sensitivity + specificity—1 in the Oxford Discovery cohort and then applied to the 2 validation groups. The age- and sex- adjusted likelihood ratio (LR) for prodromal PD was calculated using the updated Movement Disorder Society (MDS) research criteria,^1^ as detailed in eMethods 1 in Supplement 1. A positive LR was defined as a probability threshold of 80% or more. Within each cohort, *t* test or 1-way analysis of variance, was used to compare age and Pearson χ^2^ to compare sex frequency between groups. L1EV α-synuclein concentrations were log-transformed to improve the distribution in group comparisons using linear regression models. Age and sex were included as covariates in linear regression models to assess these demographic differences in L1EV α-synuclein across study populations; additional covariates were used when applicable. Correlations between biomarkers were calculated using Pearson correlation when data were normally distributed or Spearman correlation when data were not normally distributed. Values with *P* < .05 were regarded as significant. The robust regression and outlier removal method was applied to test for outliers. Statistical analyses were performed using SPSS version 25.0 (IBM) and Prism version 9.4 (GraphPad).

### Data Availability

Anonymized individual participant data and the study protocol will be shared with qualified parties on request to the corresponding author. Clinical data for the PPMI cohort should be requested via the PPMI portal.^17^

## Results

### Demographic Characteristics

Among 576 participants included, the mean (SD) age was 64.30 (8.27) years, 394 were male (68.4%), and 182 were female (31.6%), as summarized in the Table. In the derivation group (Oxford Discovery), 170 participants (mean [SD] age, 64.18 [9.88] years; 156 male [91.8%] and 14 female [8.2%]) were included. In the first validation group (Marburg+Cologne), 122 participants (mean [SD] age, 65.19 [8.18] years; 91 male [74.6%] and 31 female [25.4%]) were included. In the second validation group (PPMI), 263 participants (mean [SD] age, 63.78 [7.30] years; 134 male [51.0%] and 129 female [49.0%]) were included. There was no effect of age or sex on L1EV α-synuclein in the prodromal groups, as summarized in eTable 1 and eFigure 3 in Supplement 1.

**Table.** Demographic Characteristics of Each Cohort^a^

Characteristic	HC^b^	iRBD	GBA1*^N409S^* NMC	Hyposmics	*P* value	Sporadic PD	GBA1*^N409S^* PD
**Oxford Discovery**							
No. of individuals	73	97	NA	NA	NA	NA	NA
Sex, No. (%)							
Female	9 (12.3)	5 (5.2)	NA	NA	.09	NA	NA
Male	64 (87.7)	92 (94.8)					
Age, mean (SD), y	63.47 (12.19)	64.57 (8.38)	NA	NA	.56	NA	NA
MoCA, mean (SD)	26.54 (2.21)	26.05 (2.55)	NA	NA	.26	NA	NA
L1EV α-syn, median (IQR), pg/mL	12.89 (7.09)	23.11 (12.03)	NA	NA	<.001	NA	NA
**Marburg**							
No. of individuals	29	51	NA	NA	NA	NA	NA
Sex, No. (%)							
Female	10 (34.5)	3 (5.9)	NA	NA	<.001	NA	NA
Male	19 (65.5)	48 (94.1)					
Age, mean (SD), y	58.32 (8.24)	67.79 (7.03)	NA	NA	<.001	NA	NA
MoCA, mean (SD)	28.17 (2.14)	27.41 (2.19)	NA	NA	.43	NA	NA
L1EV α-syn, median (IQR), pg/mL	11.55 (4.31)	29.06 (15.08)	NA	NA	<.001	NA	NA
**Cologne**							
No. of individuals	18	24	NA	NA	NA	NA	NA
Sex, No. (%)							
Female	15 (83.3)	3 (13.6)	NA	NA	<.001	NA	NA
Male	3 (16.7)	21 (86.4)					
Age, mean (SD), y	66.69 (7.03)	66.90 (6.81)	NA	NA	.92	NA	NA
MoCA, mean (SD)	27.61 (2.45)	27.21 (1.84)	NA	NA	.55	NA	NA
L1EV α-syn, median (IQR), pg/mL	10.84 (9.31)	21.39 (16.41)	NA	NA	.002	NA	NA
**PPMI**							
No. of individuals	20	27	146	20		50	21
Sex, No. (%)							
Female	9 (45.0)	5 (18.5)	91 (62.3)	4 (20.0)	<.001	20 (40.0)	8 (38.1)
Male	11 (55.0)	22 (81.5)	55 (37.7)	16 (80.0)		30 (60.0)	13 (61.9)
Age, mean (SD), y	61.27 (10.66)	68.13 (5.44)	62.47 (6.74)	70.59 (6.61)	<.001	63.55 (6.07)	66.47 (7.19)
MoCA, mean (SD)	28.00 (1.30)	26.37 (3.71)	26.94 (2.34)	27.10 (1.86)	.21	26.48 (2.21)	26.14 (3.04)
L1EV α-syn, (median [IQR]), pg/mL	15.61 (8.35)	24.52 (19.93)	16.08 (16.14)	20.51 (24.18)	<.001	26.55 (33.24)	26.94 (27.44)

Abbreviations: α-syn, α-synuclein; iRBD, isolated REM Sleep Behavior Disorder; GBA1, glucocerebrosidase; HC, healthy controls; L1EV, L1CAM positive extracellular vesicles; MoCA, Montreal Cognitive Assessment; NA, not applicable; NMC, nonmanifest carriers; PD, Parkinson disease; PPMI, Parkinson’s Progression Markers Initiative.

^a^ Within each cohort, *t* test or 1-way analysis of variance was used to compare age and MoCA, Mann-Whitney *U* test or Kruskal-Wallis 1-way analysis of variance to compare L1EV α-Syn, and Pearson χ^2^ test to compare sex frequency between groups.

^b^ MoCA was not available for 40 controls.

### α-Synuclein in Serum L1EV is Elevated in Participants With iRBD Across Single-Center Cohorts

**Figure 1.**
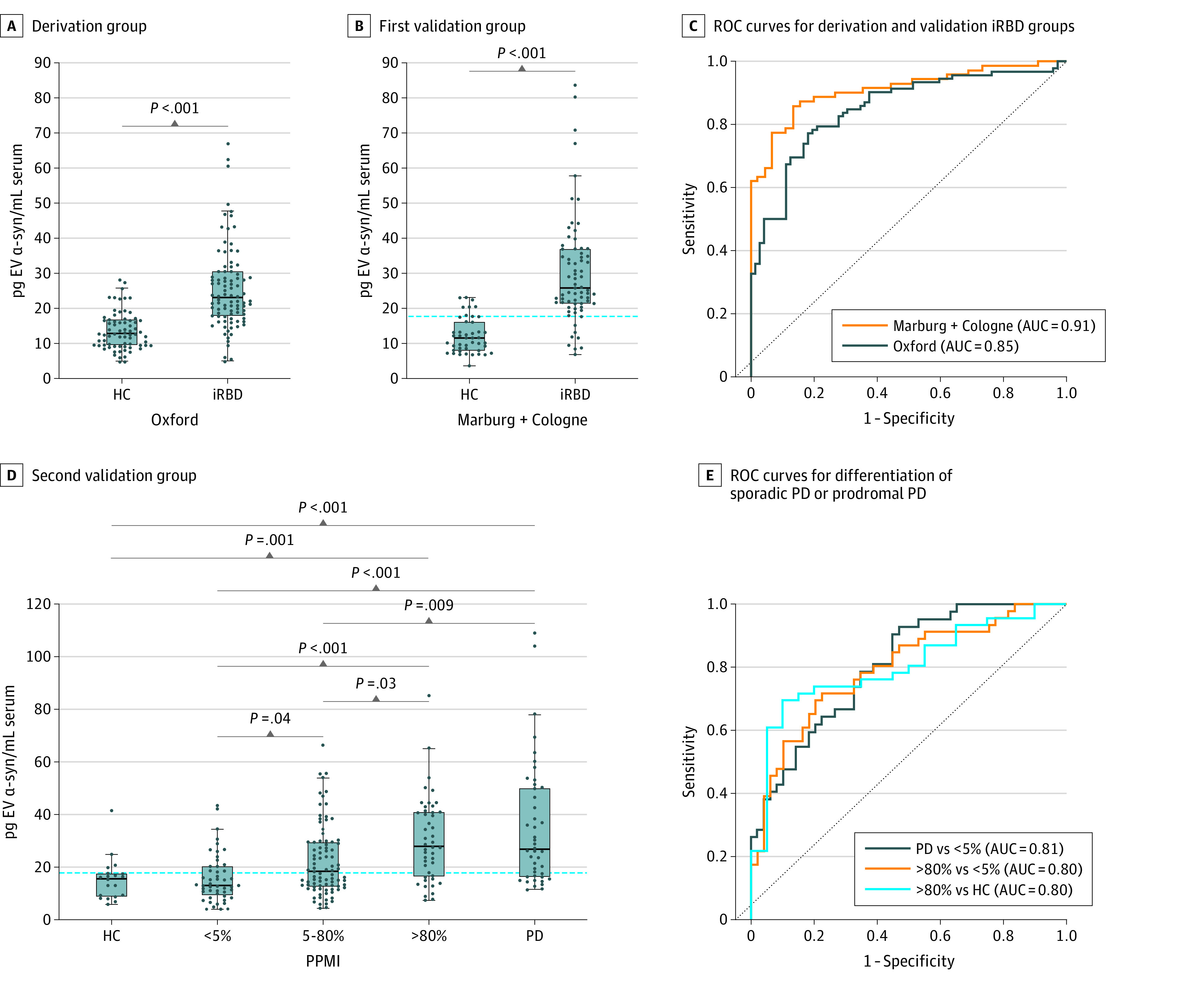
L1CAM Extracellular Vesicle (L1EV) α-Synuclein Levels in the Derivation and Validation Groups Boxplots showing the distribution of L1EV α-synuclein in (A) the derivation group (Oxford Discovery rapid eye movement sleep behavior disorder [iRBD] cohort) and (B) the first validation group (Marburg+Cologne iRBD cohorts) when compared with controls. The derived threshold of 17.75 pg/mL was applied to the first validation group, showing the consistency of the biomarker across iRBD cohorts. (C) Corresponding receiver operating characteristic (ROC) curves for the derivation and validation iRBD groups. (D) The derived threshold of 17.75 pg/mL also distinguished participants with more than 80% probability of having prodromal Parkinson disease (PD) who exhibited increased L1EV α-synuclein levels compared with those with minimal or intermediate risk in the second validation group (Parkinson’s Progression Markers Initiative [PPMI] cohort). Healthy controls and participants with sporadic PD were included as reference groups. (E) Corresponding ROC curves for the differentiation of participants with sporadic PD or more than 80% probability of having prodromal PD from healthy controls or those with less than 5% probability of having prodromal PD. The midline of the box plots indicates the median and the box indicates the 25th and 75th percentiles. Outliers and whiskers were plotted using the Tukey method. Statistical significance was determined by Mann-Whitney or Kruskal-Wallis test. AUC indicates area under the ROC curve; α-syn, α-synuclein; HC, healthy controls.

Deeply phenotyped participants with iRBD were studied initially to determine whether L1EV α-synuclein is increased in this prodromal group who have the highest risk of converting to PD or DLB compared with controls. It was confirmed (data shown as median with IQR) that L1EV α-synuclein is elevated in the Oxford Discovery iRBD cohort that was studied previously^7^ by analyzing serum samples from 97 participants with iRBD and 73 controls. L1EV α-synuclein levels were consistent in iRBD (23.11 [IQR, 12.03] pg/mL) when compared with what was previously reported for a subgroup of this cohort (n = 56; 22.17 [IQR, 14.31] pg/mL),^7^ which were increased when compared with controls (12.89 [IQR, 7.09] pg/mL; *P* < .001) as shown in Figure 1A. This finding was further confirmed in participants with iRBD from Marburg+Cologne consisting of 75 cases and 47 controls (Figure 1B). These analyses showed that L1EV α-synuclein at a threshold of 17.75 pg/mL (derived from Oxford Discovery) exhibited a consistent performance in the independent iRBD group (Marburg+Cologne), as shown in Figure 1C, and for each of these cohorts in eFigure 4 in Supplement 1. In the derivation group, AUC = 0.85 (95% CI, 0.79-0.91) with sensitivity of 0.77 (95% CI, 0.68-0.85) and specificity of 0.82 (95% CI, 0.72-0.89). In the validation group, AUC = 0.91 (95% CI, 0.86-0.96) with sensitivity of 0.87 (95% CI, 0.78-0.93) and specificity of 0.82 (95% CI, 0.69-0.91). Overall, there was an approximately 2-fold increase in L1EV α-synuclein levels across the participants with iRBD (24.21 [IQR, 13.75] pg/mL) compared with the controls (12.20 [IQR, 7.39] pg/mL) with AUC = 0.88 (95% CI, 0.84-0.92).

### Serum L1EV α-Synuclein Levels Differentiate Those With Probable Prodromal PD from Participants With Minimal Risk in a Multicenter Cohort

Having established the consistency of the biomarker in a fairly homogeneous group of high-risk participants, the broader applicability of the derived threshold from Oxford Discovery was investigated in a multicenter cohort (PPMI) consisting of a heterogenous group with variable risk of developing PD or related dementia, including *GBA1^N409S^* gene carriers (n = 146) and participants with hyposmia (n = 20) or iRBD (n = 27). Healthy controls (n = 20) and patients with sporadic PD (n = 50) from PPMI were included as negative and positive reference groups, respectively. For this analysis, the age- and sex-adjusted probability of having prodromal PD was calculated for each participant using updated MDS prodromal research criteria^1^ detailed in eMethods 1 in Supplement 1. Participants were stratified into those with less than 5% probability (n = 57), those with 5% to 80% probability (n = 89), and those with a positive lifetime risk, which is defined as more than 80% probability (n = 47), as shown in eTable 2 in Supplement 1. Participants with more than 80% probability of having prodromal PD exhibited increased L1EV α-synuclein levels (27.93 [IQR, 23.80] pg/mL) similar to patients with sporadic PD (26.55 [IQR, 33.24] pg/mL), whereas participants with less than 5% probability of having prodromal PD exhibited L1EV α-synuclein levels (12.98 [IQR, 10.32] pg/mL) similar to healthy controls (15.61 [IQR, 8.35] pg/mL). L1EV α-synuclein levels in participants with 5% to 80% probability of having prodromal PD (18.13 [IQR, 16.33] pg/mL) were intermediate between participants with more than 80% and participants with less than 5% probability of having prodromal PD or healthy controls, as shown in Figure 1D. By applying the threshold derived from the Oxford Discovery cohort (17.75 pg/mL), participants with more than 80% probability of having prodromal PD were differentiated from those with less than 5% risk with an AUC of 0.80 (95% CI, 0.71-0.89), sensitivity of 0.74 (95% CI, 0.60-0.84), and specificity of 0.67 (95% CI, 0.53-0.79) and from controls with an AUC of 0.80 (95% CI, 0.69-0.91), sensitivity of 0.74 (95% CI, 0.60-0.84), and specificity of 0.80 (95% CI, 0.58-0.92), as shown in Figure 1E. Similarly, patients with sporadic PD were differentiated from those with less than 5% risk with an AUC of 0.81 (95% CI, 0.72-0.89) and from controls with an AUC of 0.81 (95% CI, 0.70-0.92). The optimal sensitivity and specificity for each subgroup is shown in eTable 3 in Supplement 1.

### L1EV α-Synuclein is Increased in Participants With Specific Additional Prodromal Markers Within At-Risk Groups and May Precede Dopaminergic Neurodegeneration

The overall estimated risk of lifelong phenoconversion for *GBA1^N409S^* gene carriers to PD is 6% to 10% by the age of 70 years.^3,18^ Therefore, *GBA1^N409S^* gene carriers may be at the earliest end of the prodromal phase. To investigate the value of L1EV α-synuclein in the stratification of this group, those *GBA1^N409S^* gene carriers were identified who exhibited any prodromal markers based on the MDS research criteria^1^ beyond their genetic status. A *GBA1^N409S^* PD group was included for reference. *GBA1^N409S^* gene carriers with constipation (n = 41; 21.21 [IQR, 25.17]; *P* = .01), urinary dysfunction based on the Unified Parkinson's Disease Rating Scale I (n = 53; 20.37 [IQR, 16.74]; *P* = .02), an MDS Unified Parkinson's Disease Rating Scale III score of more than 6 excluding postural or action tremor (n = 12; 27.26 [IQR, 23.26]; *P* = .02), or cognitive deficit based on Montreal Cognitive Assessment score less than 26 (n = 33; 18.13 [IQR, 16.61]; *P* = .03) exhibited higher L1EV α-synuclein as shown in eTable 4 and eFigure 5 in Supplement 1.

The association between prodromal markers and L1EV α-synuclein levels in participants with iRBD with available relevant assessments (n = 127) was then assessed. Because iRBD conveys a much higher risk of prodromal PD than *GBA1* gene mutations,^1^ all of the participants with iRBD had additional prodromal markers. To identify the main contributors in this group, multiple linear regression analysis was performed. Hyposmia (β = 0.178 [standard error (SE), 0.065]; *P* = .01) and cognitive deficit (β = 0.116 [SE, 0.052]; *P* = .03) exhibited the strongest association with L1EV α-synuclein levels as shown eTable 5 in Supplement 1.

**Figure 2.**
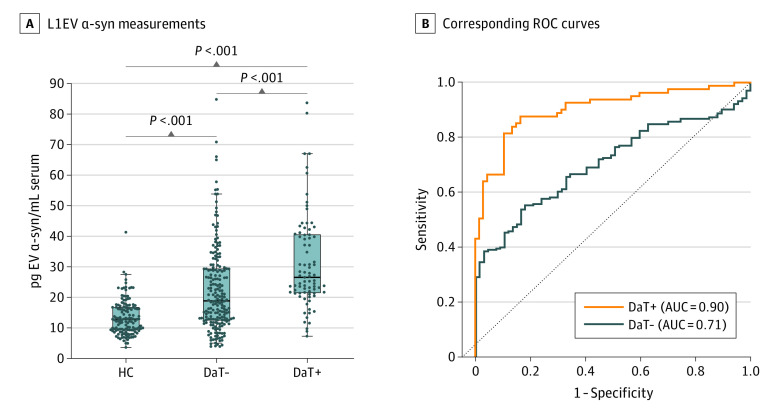
L1CAM Extracellular Vesicle (L1EV) α-Synuclein Measurements in At-Risk Participants With Available Dopamine Transporter Single-Photon Emission Computed Tomography (A) Boxplot of L1EV α-synuclein measurements and (B) corresponding ROC curves for healthy controls (HC) and at-risk participants with a negative/normal (DaT-) or positive/abnormal (DaT+) dopamine transporter single-photon emission computed tomography. The midline of the box plots indicates the median and the box indicates the 25th and 75th percentiles. Outliers and whiskers were plotted using the Tukey method. Statistical significance was determined by Kruskal-Wallis test. AUC indicates area under the receiver operating characteristic curve; α-syn, α-synuclein; ROC, receiver operating characteristic.

Lastly, the biomarker was assessed in relation to the presence of dopaminergic neurodegeneration, as documented in participants with available DaT SPECT. Nuclear imaging is the only available test to definitively demonstrate dopaminergic nerve loss in life. An abnormal DaT SPECT signifies progression of pathology and a higher risk of conversion to PD in participants with iRBD or hyposmia.^2,19,20,21^ Forty seven iRBD participants from Oxford, 50 participants with iRBD from Marburg, 22 participants with iRBD from Cologne, 27 participants with iRBD, 20 hyposmic participants, and 141 *GBA1^N409S^* gene carriers from the PPMI cohort had available DaT SPECT results (n = 307). In this comparison, L1EV α-synuclein levels were highest in participants with an abnormal DaT SPECT (26.55 [IQR, 18.31] pg/mL), intermediate in those with a normal DaT SPECT (18.92 [IQR, 14.57] pg/mL), and lowest in healthy controls (12.60 [IQR, 6.68] pg/mL), as shown in Figure 2A. ROC revealed an improved differentiation from controls for those with abnormal DaT SPECT (AUC = 0.90; 95% CI, 0.86-0.95) compared with those with normal DaT SPECT (AUC = 0.71; 95% CI, 0.65-0.76) as shown in Figure 2B. This trend was seen in each subgroup (*GBA1^N409S^* gene carriers, iRBD, and hyposmics) as shown in eFigure 6 in Supplement 1. There was no correlation between L1EV α-synuclein levels and Montreal Cognitive Assessment or subthreshold parkinsonism scores when analyzed across each condition (eFigure 7 in Supplement 1) or each cohort (eTable 6 in Supplement 1).

### L1EV α-Synuclein Differentiates Those At Risk of Developing PD From Healthy Control Populations Irrespective of Initial Diagnosis

**Figure 3.**
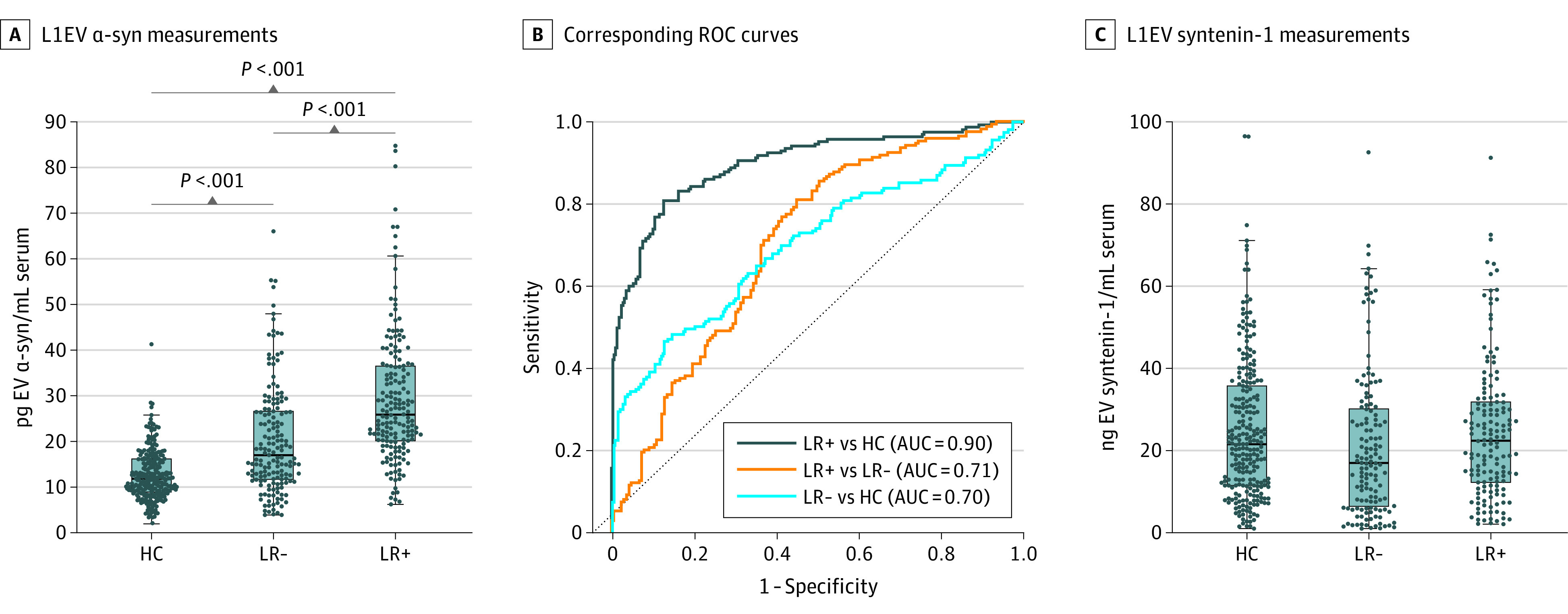
L1CAM Extracellular Vesicle (L1EV) α-Synuclein Levels Are Increased in At-Risk Participants With Positive Likelihood Ratio (LR) of Developing Parkinson Disease (PD) (A) Boxplot of L1EV α-synuclein (α-syn) measurements and (B) corresponding receiver operating characteristic (ROC) curves for those with positive likelihood ratio (LR+) or negative likelihood ratio (LR-) of developing PD based on the updated Movement Disorder Society prodromal research criteria or current and historic healthy controls (HC). (C) Boxplot showing measurements for L1EV syntenin-1, which is a generic extracellular vesicles-associated marker. The midline of the box plots indicates the median and the box indicates the 25th and 75th percentiles. Outliers and whiskers were plotted using the Tukey method. Statistical significance was determined by Kruskal-Wallis test.

L1EV α-synuclein was assessed across all at-risk participants with any available prodromal markers that are shown in eTable 7 in Supplement 1. One hundred and ninety participants who fulfilled the proposed criteria for prodromal PD (ie, probability more than 80%, LR positive) had higher serum L1EV α-synuclein levels compared with 175 participants with a negative LR, as shown in Figure 3A. L1EV α-synuclein levels were also compared in LR positive and LR negative participants to the current (12.60 [IQR, 6.68] pg/mL) and historic (11.12 [IQR, 6.23] pg/mL) controls (eFigure 8 in Supplement 1) from multiple cohorts^7,8^ (282 in total) to obtain as accurate values as possible that would be applicable across populations. L1EV α-synuclein discriminated LR positive participants from controls with AUC of 0.90 (95% CI, 0.87-0.93), sensitivity of 0.81 (95% CI, 0.75-0.86), and specificity of 0.87 (95% CI, 0.83-0.91) and was less informative in LR negative participants to controls, as shown in Figure 3B and eTable 8 in Supplement 1. There were no consistent differences in the generic EV marker syntenin-1 across these groups (Figure 3C; eFigure 9 in Supplement 1).

### Association of Serum L1EV α-Synuclein Levels With CSF Pathology and Phenoconversion

**Figure 4.**
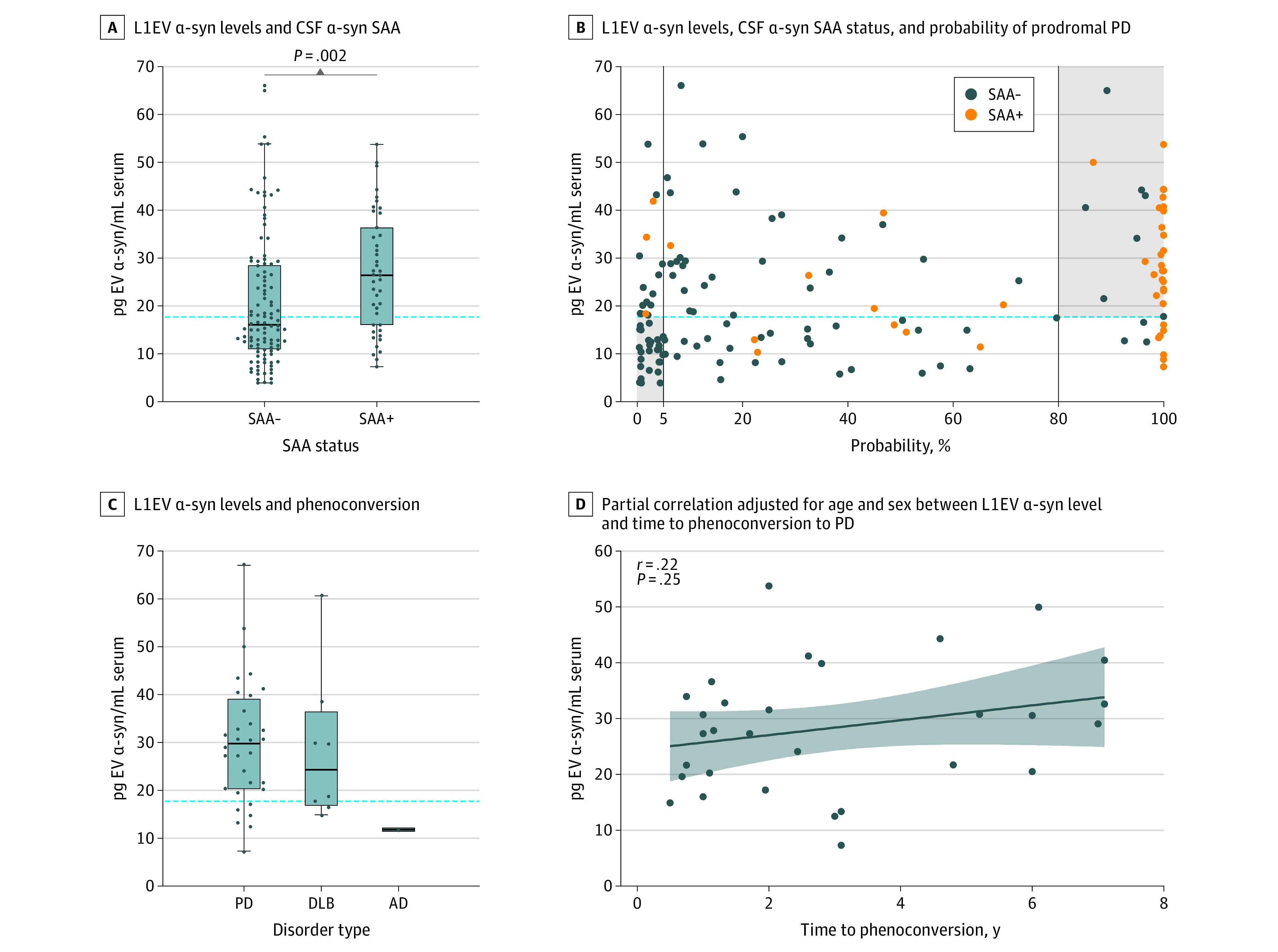
L1CAM Extracellular Vesicle (L1EV) α-Synuclein Levels and Cerebrospinal Fluid (CSF) Seed Amplification Assay (SAA) or Phenoconversion (A) Serum L1EV α-synuclein (α-syn) levels were higher in participants with positive CSF α-synuclein SAA. The midline of the box plots indicates the median and the box indicates the 25th and 75th percentiles. The horizontal dotted line represents the derived threshold (17.75 pg/mL) of L1EV α-synuclein. Outliers and whiskers were plotted using the Tukey method. Statistical significance was determined by Mann-Whitney test. (B) Association between serum L1EV α-synuclein levels, CSF α-synuclein SAA status, and probability of having prodromal Parkinson disease (PD). The horizontal dotted line represents the derived threshold (17.75 pg/mL) of L1EV α-synuclein. The vertical lines represent the age and sex adjusted probability of having prodromal PD at less than 5% and more than 80%. (C) Boxplot of serum L1EV α-synuclein levels in at-risk participants who phenoconverted to PD (n = 32), dementia with Lewy bodies (DLB) (n = 8), and Alzheimer disease (AD) (n = 1). The horizontal dotted line represents the derived threshold (17.75 pg/mL) of L1EV α-synuclein. The midline of the box plots indicates the median and the box indicates the 25th and 75th percentiles. Outliers and whiskers are plotted using the Tukey method. (D) Partial correlation adjusted for age and sex between serum L1EV α-synuclein levels and time to phenoconversion to PD. Least squares regression line with 95% CI was plotted and Pearson coefficient was reported.

CSF α-synuclein SAA, which signifies the presence of brain pathology, was recently shown to detect a subgroup of at-risk participants in the prodromal phase in the PPMI cohort.^22^ To investigate the relevance of the biomarker to this readout of pathology, SAA results for the PPMI participants with hyposmia or iRBD or *GBA1^N409S^* (n = 157) in relation to L1EV α-synuclein levels and their probability of having prodromal PD were utilized. Those with positive CSF SAA had higher L1EV α-synuclein in serum (26.41 [IQR, 19.54] pg/mL) compared with those who were CSF SAA negative (15.88 [IQR, 16.11] pg/mL), as shown in Figure 4A and eFigure 10 in Supplement 1. The SAA positive group primarily included those participants with more than 80% probability of having prodromal PD who were also above the identified threshold derived from Oxford Discovery, as shown in Figure 4B. These data show that 68.3% of SAA negative low-risk participants (less than 5% probability) were also below the L1EV α-synuclein threshold derived from Oxford Discovery, whereas 75.9% of SAA positive, high-risk participants (more than 80% probability), were above the threshold (Figure 4B). In the intermediate group (5% to 80% risk), 43.3% of SAA negative were below the L1EV α-synuclein threshold and 50% of SAA positive were above the threshold (Figure 4B).

Lastly, across all cohorts 8 cases who converted to DLB, 32 cases who converted to PD, and 1 case who converted to Alzheimer disease (AD) were identified based on clinical diagnoses (Figure 4C). Thirty two of the 40 cases (80% overall; 81.3% of PD; 75% of DLB) that phenoconverted to neuronal α-synucleinopathy were above the threshold of 17.75 pg/mL, whereas the single Alzheimer disease case had L1EV α-synuclein below the threshold. In these clinically diagnosed cases, L1EV α-synuclein was lower in DLB cases (24.32 [IQR, 14.53] pg/mL) than in the PD cases (30.59 [IQR, 17.85] pg/mL) in line with the initial report in neuropathologically confirmed DLB cases.^7^ In this small number of cases, there was a weak partial correlation after correcting for age and sex (r = 0.22; *P* = .25) between the levels of L1EV α-synuclein and time to phenoconversion (Figure 4D). There was also a weak inverse correlation between serum L1EV α-synuclein and total CSF α-synuclein in those participants who phenoconverted to PD and a significant inverse correlation in those with sporadic PD (eFigure 11 in Supplement 1). These data suggest that high α-synuclein in L1EVs is associated with lower total α-synuclein in CSF and longer time to conversion to PD.

## Discussion

In this study, we showed that L1EV α-synuclein levels are increased in participants at risk of developing PD and related dementia. Participants with iRBD exhibited a 2-fold increase in L1EV α-synuclein levels compared with controls as validated across independent iRBD groups. This is in line with our previous estimate of approximately 2-fold increase in L1EV α-synuclein levels across multiple cohorts of sporadic PD vs controls^7,8^ and consistent with the idea that both conditions are α-synucleinopathies. Furthermore, a threshold derived from the largest iRBD group also differentiated participants with more than 80% probability of having prodromal PD from those with low risk in a further independent multicenter validation group (PPMI). This validation group consists of participants with variable risk recruited across multiple sites in the US, Europe, and Israel that could explain the slightly reduced performance of the biomarker in this group. Nevertheless, our findings demonstrate the robustness of the assay as a prodromal biomarker of α-synucleinopathy irrespective of recruitment site or specific diagnosis. Taken together with our previous reports that L1EV α-synuclein levels are increased in PD and DLB but not MSA,^7,8^ the biomarker offers a blood-based measurement to identify prodromal neuronal α-synucleinopathy. This conclusion is further reinforced in those participants who phenoconverted to PD and DLB as 80% were correctly identified by the biomarker.

Across cohorts, subgroups with more than 1 prodromal marker exhibited higher L1EV α-synuclein levels. Thus, measurements of L1EV α-synuclein in combination with specific prodromal markers, such as olfactory or cognitive deficit, possible or definite RBD or *GBA1* gene status could be harnessed to aid the substratification of those at-risk individuals with the highest probability of developing PD and related Lewy body diseases. Additive risk was previously observed with other combination markers, such as hyposmia, age, constipation, and abnormal DaT SPECT.^21^ Measurement of L1EV α-synuclein could offer a cheaper and more accessible alternative means of screening at-risk individuals compared with a DaT SPECT or labor-intensive clinical assessments. L1EV α-synuclein levels are also increased in DaT SPECT negative at-risk participants, indicating that α-synuclein dyshomeostasis may be detectable in blood before dopaminergic neurodegeneration.

L1EV α-synuclein levels were higher in participants with a positive CSF SAA; 76% of those participants with a positive CSF SAA and more than 80% probability of having prodromal PD had L1EV α-synuclein above the threshold. Although none of these cases are neuropathologically confirmed, our data suggest that serum L1EV α-synuclein is a proxy biomarker of pathology based on this CSF assay, as we previously found in a small number of neuropathologically confirmed DLB cases.^7^ It should be noted that those participants with L1EV α-synuclein above the threshold but with negative CSF SAA in the intermediate group are still at-risk participants based on probability scores and at this stage, we do not know for certain whether egress of α-synuclein in serum L1EV precedes pathology that is detectable by CSF SAA. Positive CSF SAA was reported in 86% of participants with iRBD or hyposmia,^22^ which is similar to the sensitivity of our blood-based assay in these groups. SAA of α-synuclein aggregates immunoprecipitated from serum was also positive in PD but only in 44% of iRBD.^23^ The latter suggests that at least in blood, egress of α-synuclein in L1EVs which was above the threshold in 80.4% (160 of 199) of our iRBD cases may precede detection or formation of aggregates in the prodromal phase. Because L1CAM is primarily expressed in neurons of both the central and peripheral nervous system, we cannot exclude the possibility that L1EVs are partly derived from postganglionic autonomic nerves that are pathologically affected early in PD and related dementia. It would be interesting to investigate in the future whether skin SAA that was recently reported in iRBD^24^ better correlates with our biomarker.

Because EVs are derived from within the cells and α-synuclein is targeted to lysosomes^25^ and released in EVs in response to lysosome inhibition,^26^ our data in patients suggest that increased L1EV secretion may reflect a fundamental defect in intracellular α-synuclein trafficking that occurs several years before the clinical presentation. This mechanism would be consistent with our finding of an inverse correlation between L1EV α-synuclein and total CSF α-synuclein in those who went on to develop PD or had sporadic PD. We also observed that in those at-risk participants who developed PD, higher L1EV α-synuclein at baseline was associated with longer interval to phenoconversion. One explanation for these observations is the existence of an adaptive, likely protective, efflux of α-synuclein outside neuronal tissues in response to defective intraneuronal processing by lysosomes, eg, due to *GBA1 gene* mutations or polymorphisms in other lysosomal genes identified by genome-wide association studies in sporadic PD. Our assay measures total α-synuclein and L1EVs may contain both physiological and pathogenic forms such as α-synuclein oligomers. For example, α-synuclein conformers with fibrillar appearance were amplified from plasma-derived L1EVs isolated from patients with PD but not controls.^27^ Therefore, amplification assays or immunoassays with conformation specific antibodies in combination with assay automation could be applied in prodromal samples to further refine the performance of the biomarker.

### Limitations

There are limitations to our findings. This is a cross-sectional study and further validation of the biomarker in longitudinal cohorts will be an important future investigation that may identify other clinical correlates or more precise thresholds. Therefore, despite group validation across different cohorts, the clinical value of this biomarker at single-participant level for routine practice is not yet fully established and cutoff values may require further refinement ideally in neuropathologically confirmed cases when at-risk cohort participants eventually reach postmortem examination. The cohorts were composed of clinical phenotypes highly predictive of α-synuclein pathology. Analyses of subgroups within these cohorts revealed associations with specific prodromal markers despite the small subset of patients. Future studies are needed to confirm these findings and extend their applicability to wider population cohorts. Where such data were available, most of the study participants self-identified as White and we were underpowered to test differences in analyte levels across racial and ethnic groups. Therefore, the results may not generalize to other populations.

## Conclusions

In summary, our results demonstrate the value of L1EV α-synuclein in identifying participants at-risk of developing neuronal α-synucleinopathy and offer patient-based measurements that support the existence of a fundamental shift in α-synuclein homeostasis that is detectable in blood at the early stages of the pathogenic cascade.

## Notes

Supplement 1. eMethods 1. Clinical assessments eMethods 2. L1EV immunocapture and protein measurements eFigure 1. Study selection criteria and design for derivation and validation groups eFigure 2. L1EV α-synuclein levels across batches eTable 1. Influence of age or sex on L1EV α-synuclein eFigure 3. Correlation between L1EV α-synuclein and age eFigure 4. L1EV α-synuclein levels in Marburg and Cologne cohorts eTable 2. Summary of prodromal markers in validation group 2 (PPMI) eTable 3. ROC analyses in derivation and validation groups eTable 4. Difference in L1EV α-synuclein levels between GBA1N409S non-manifest carriers with and without prodromal markers eFigure 5. L1EV α-synuclein measurements in GBA1N409S gene carriers eTable 5. Association between L1EV α-synuclein and prodromal markers in iRBD eFigure 6: L1EV α-synuclein in prodromal groups with available DaT SPECT eFigure 7. Correlation between L1EV α-synuclein and cognitive or motor scores eTable 6. Correlation analyses in each cohort eTable 7. Summary of prodromal markers eFigure 8. L1EV α-synuclein comparison between current and historic controls eTable 8. ROC analyses to stratify at-risk subjects eFigure 9. L1EV-associated syntenin-1 levels in each condition eFigure 10. L1EV α-synuclein in prodromal groups with available SAA eFigure 11. Correlation between serum L1EV α-synuclein and CSF total α-synuclein eReferences

Supplement 2. Data sharing statement
