# Supplementary material for: Neuronally Derived Extracellular Vesicle α-Synuclein as a Serum Biomarker for Individuals at Risk of Developing Parkinson Disease
Source: JAMA Neurol. 2023 Dec 4;81(1):59–68. doi: 10.1001/jamaneurol.2023.4398 (PMC10696516; doi:10.1001/jamaneurol.2023.4398)
Supplement: Supplement 1. — eMethods 1. Clinical assessments eMethods 2. L1EV immunocapture and protein measurements eFigure 1. Study selection criteria and design for derivation and validation groups eFigure 2. L1EV α-synuclein levels across batches eTable 1. Influence of age or sex on L1EV α-synuclein eFigure 3. Correlation between L1EV α-synuclein and age eFigure 4. L1EV α-synuclein levels in Marburg and Cologne cohorts eTable 2. Summary of prodromal markers in validation group 2 (PPMI) eTable 3. ROC analyses in derivation and validation groups eTable 4. Difference in L1EV α-synuclein levels between GBA1N409S non-manifest carriers with and without prodromal markers eFigure 5. L1EV α-synuclein measurements in GBA1N409S gene carriers eTable 5. Association between L1EV α-synuclein and prodromal markers in iRBD eFigure 6: L1EV α-synuclein in prodromal groups with available DaT SPECT eFigure 7. Correlation between L1EV α-synuclein and cognitive or motor scores eTable 6. Correlation analyses in each cohort eTable 7. Summary of prodromal markers eFigure 8. L1EV α-synuclein comparison between current and historic controls eTable 8. ROC analyses to stratify at-risk subjects eFigure 9. L1EV-associated syntenin-1 levels in each condition eFigure 10. L1EV α-synuclein in prodromal groups with available SAA eFigure 11. Correlation between serum L1EV α-synuclein and CSF total α-synuclein eReferences [file jamaneurol-e234398-s001.pdf]

# Supplemental Online Content

Yan S, Jiang C, Janzen A, et al. Neuronally-derived extracellular vesicle α-synuclein as a serum biomarker for individuals at risk of developing Parkinson disease. *JAMA Neurol*. Published online November 20, 2023. doi:10.1001/jamaneurol.2023.4398

**eMethods 1.** Clinical assessments
**eMethods 2.** L1EV immunocapture and protein measurements
**eFigure 1.** Study selection criteria and design for derivation and validation groups
**eFigure 2.** L1EV α-synuclein levels across batches
**eTable 1.** Influence of age or sex on L1EV α-synuclein
**eFigure 3.** Correlation between L1EV α-synuclein and age
**eFigure 4.** L1EV α-synuclein levels in Marburg and Cologne cohorts
**eTable 2.** Summary of prodromal markers in validation group 2 (PPMI)
**eTable 3.** ROC analyses in derivation and validation groups
**eTable 4.** Difference in L1EV α-synuclein levels between $GBA1^{N409S}$ non-manifest carriers with and without prodromal markers
**eFigure 5.** L1EV α-synuclein measurements in $GBA1^{N4095}$ gene carriers
**eTable 5.** Association between L1EV α-synuclein and prodromal markers in iRBD
**eFigure 6:** L1EV α-synuclein in prodromal groups with available DaT SPECT
**eFigure 7.** Correlation between L1EV α-synuclein and cognitive or motor scores
**eTable 6.** Correlation analyses in each cohort
**eTable 7.** Summary of prodromal markers
**eFigure 8.** L1EV α-synuclein comparison between current and historic controls
**eTable 8.** ROC analyses to stratify at-risk subjects
**eFigure 9.** L1EV-associated syntenin-1 levels in each condition
**eFigure 10.** L1EV α-synuclein in prodromal groups with available SAA
**eFigure 11.** Correlation between serum L1EV α-synuclein and CSF total α-synuclein
**eReferences**

This supplemental material has been provided by the authors to give readers additional information about their work.

© 2023 Yan et al. *JAMA Neurol.*

## **eMethods 1.** Clinical assessments

All subjects were assessed clinically as part of on-going research programmes, including the updated prodromal MDS criteria[1] using MDS UPDRS I and III, autonomic dysfunction (SCOPA-AUT) and the Montreal Cognitive Assessment (MoCA). All iRBD were confirmed by video-assisted polysomnography. Possible RBD was defined based on RBD screening questionnaire (RBDSQ) score ≥ 5. Subthreshold parkinsonism was confirmed based on MDS-UPDRS-III >6 excluding postural and action tremor. Constipation, excessive daytime somnolence, symptomatic OH, urinary dysfunction, and depression was defined by MDS UPDRS I. Erectile dysfunction was determined based on SCOPA-AUT. Cognitive deficit was defined as MoCA score <26. Hyposmia was defined as threshold/discrimination/identification (TDI) score ≤26 in the Marburg cohort[2], identification scores below the $10^{th}$ centile (9 for age >55 and 12 for age between 36-55) for Oxford and Cologne or <15% University of Pennsylvania Smell Identification Test (UPSIT) corrected for age and sex, for PPMI. Dopamine Transporter (DaT) SPECT imaging was acquired using standard protocol. Classification of SPECT imaging as normal or abnormal was based on the descriptive reports by an expert Nuclear Medicine Radiologist, who was blinded to all clinical data other than age and sex or previously described quantitative measures. The age- and sex- adjusted likelihood ratio (LR) for prodromal PD was calculated using the updated Movement Disorder Society (MDS) research criteria.[1] This was done by multiplying all available LR for each of the prodromal marker as recommended by the MDS task force. Total LR was then combined with baseline estimated age-adjusted probability to calculate the final post-test probability for each individual. A positive LR was defined as a probability threshold of ≥80%.

© 2023 Yan et al. *JAMA Neurol.*

## **eMethods 2.** L1EV immunocapture and protein measurements

Blood samples from all the centres were collected during the patient assessment, the serum was isolated, aliquoted and frozen at -80 ˚C until further use. All samples were sent on dry ice and processed in a blinded fashion at Oxford. We used our previously developed and extensively validated[3, 4] direct immunocapture assay to isolate L1EVs from 250 μl of serum. Electrochemiluminescence was performed in 96-well Meso Scale Discovery (MSD) U-Plex plates. Antibody pairs for α-synuclein were provided by MSD and pre-conjugated with biotin and ruthenium tag: For capture, the assay utilises the rabbit monoclonal antibody MJFR1 (Abcam) which binds to α-synuclein amino acids 118-123 with similar binding to monomeric or aggregated forms of α-synuclein[5]; for detection, an MSD proprietary mouse monoclonal antibody was used that binds between amino acids 15-125 with similar binding for monomers and oligomers. This antibody pair has been cross-validated for the detection of total plasma α-synuclein against other immunoassay platforms[6]. The MJFR1 antibody was previously validated by us for the detection of serum L1EV α-synuclein using electrochemistry.[7] Additive-free anti-syntenin-1 (goat) polyclonal antibody (PAB7132, Abnova) and anti-syntenin-1 (rabbit) monoclonal antibody (ab236071, Abcam) were conjugated with biotin and ruthenium and used as capture and detection antibodies respectively.

© 2023 Yan et al. *JAMA Neurol.*

**eFigure 1.** Study selection criteria and design for derivation and validation groups.

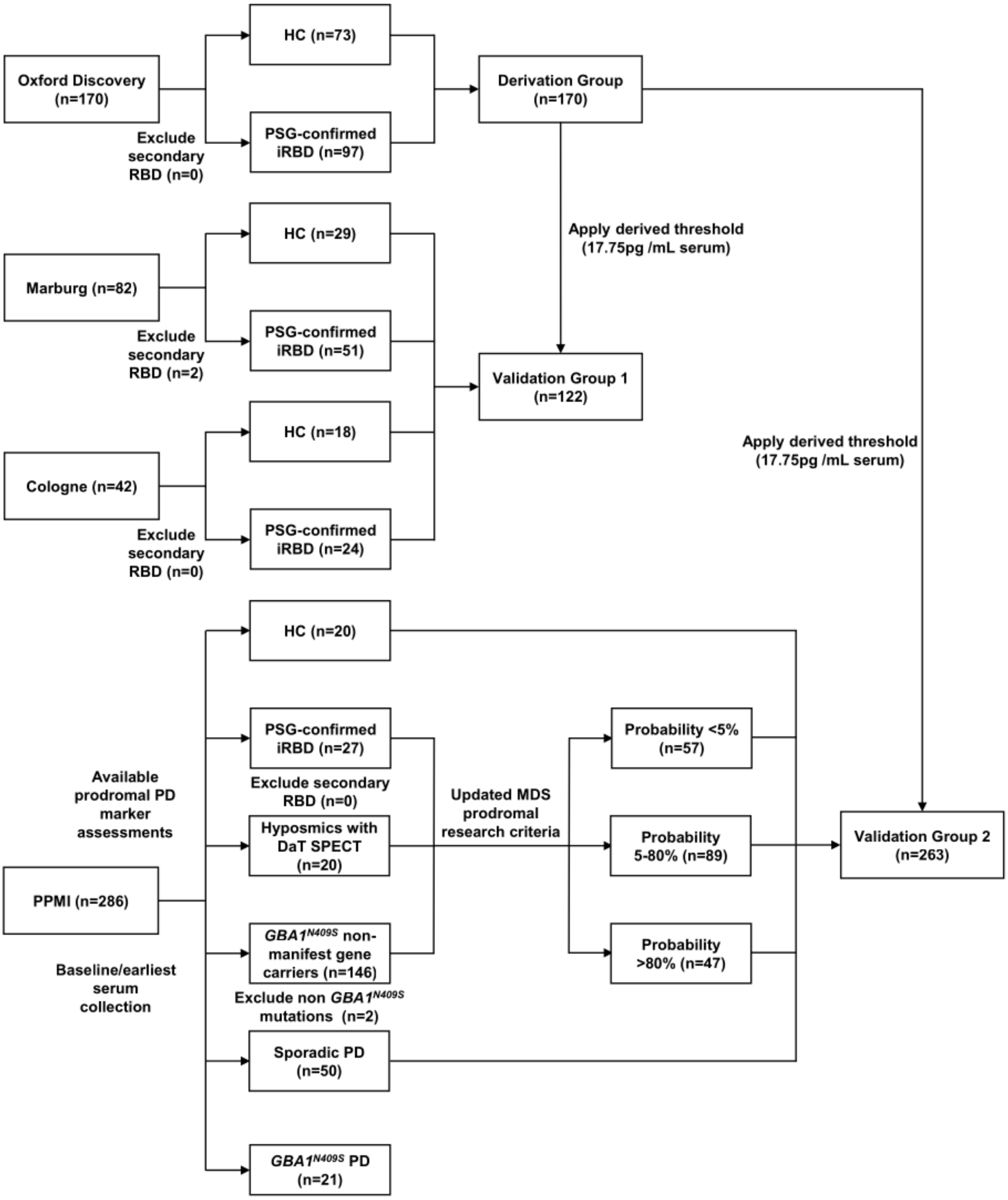


Subjects from the four cohorts were divided into one Derivation group and two Validation groups to assess whether L1EV α-synuclein levels distinguish those with the highest risk of developing PD and related dementia (i.e. either iRBD or >80% probability based on the updated MDS prodromal research criteria) from healthy controls or those with minimal risk.

**eFigure 2.** L1EV α-synuclein levels across batches.

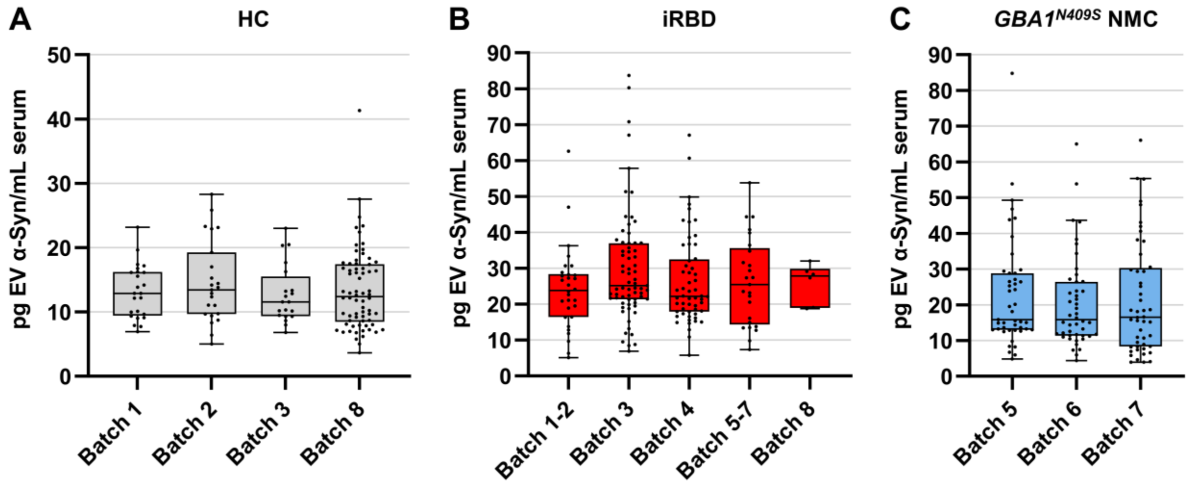


The samples were processed by operators who were blinded to the diagnosis. Following sample processing and unblinding, we compared L1EV α-synuclein levels across batches in **(A)** controls, **(B)** iRBD subjects and **(C)** *GBA1*$^{N409S}$ non-manifest gene carriers (NMC). Batch 1, 2 and 4= Oxford; Batch 3= Marburg and Cologne; Batch 5=PPMI plate 1; Batch 6=PPMI plate 2; Batch 7=PPMI plate 3; Batch 8= Oxford, Marburg, Cologne and PPMI. The midline of the box plots indicates the median, and the box indicates the 25th and 75th percentiles. Outliers and whiskers are plotted using the Tukey method. Statistical significance was determined by Kruskal-Wallis test.

© 2023 Yan et al. *JAMA Neurol.*

**eTable 1.** Influence of age or sex on L1EV α-synuclein.

| | iRBD (n=199) | | | *GBA1*$^{N409S}$ NMC (n=146) | | | Hyposmics (n=20) | | | HC (n=127) | | |
|---|---|---|---|---|---|---|---|---|---|---|---|---|
| **Variable** | **β** | **SE** | **p-value** | **β** | **SE** | **p-value** | **β** | **SE** | **p-value** | **β** | **SE** | **p-value** |
| Intercept | 1.209 | 0.136 | <.001 | 1.202 | 0.276 | <.001 | 0.896 | 0.774 | 0.262 | 1.141 | 0.107 | <.001 |
| Age | 0.003 | 0.002 | .17 | 0.001 | 0.004 | .80 | 0.007 | 0.011 | 0.513 | -0.0001 | 0.002 | 0.93 |
| Sex [Female] | -0.023 | 0.058 | .70 | 0.028 | 0.061 | .64 | -0.021 | 0.140 | 0.884 | -0.042 | 0.039 | 0.29 |
| Model R-square | .011 | | | .002 | | | .027 | | | .010 | | |
| Model p-value | .37 | | | .86 | | | .79 | | | 0.57 | | |

Multiple linear regression within each group (iRBD, *GBA1*$^{N409S}$ non-manifest gene carriers (NMC), hyposmics and healthy controls, HC), did not reveal an association between the biomarker and sex or age using the equation log (Biomarker) = $\beta_0$ + ($\beta_1$ × Age) + ($\beta_2$ × Sex) + ε.

© 2023 Yan et al. *JAMA Neurol.*

**eFigure 3.** Correlation between L1EV α-synuclein and age.

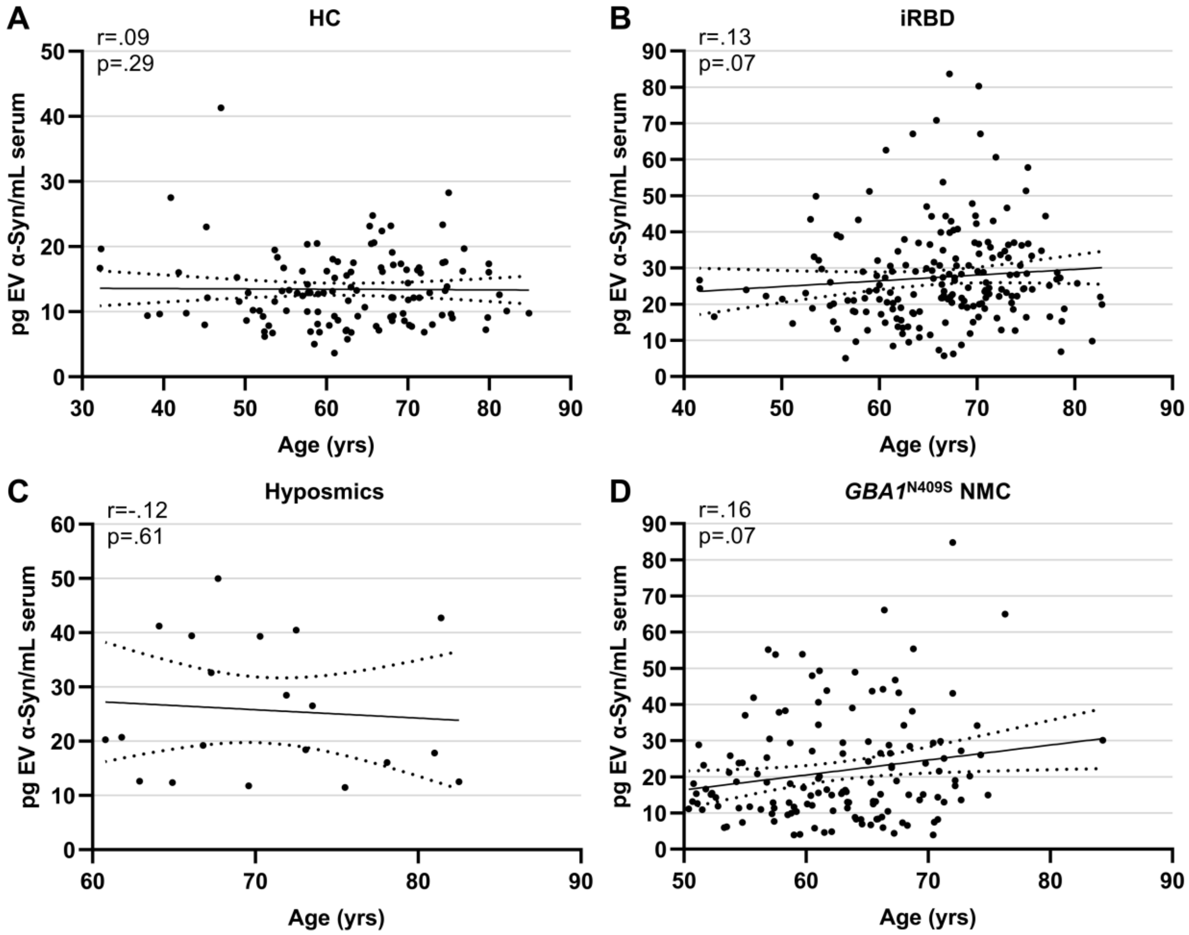


There was no significant correlation between L1EV α-synuclein levels and age at sampling in any of the four groups (healthy controls, iRBD, *GBA1*$^{N409S}$ non-manifest gene carriers (NMC)). Least squares regression line with 95% CI was plotted and Spearman coefficient with p value was reported for each group.

**eFigure 4.** L1EV α-synuclein levels in Marburg and Cologne cohorts.

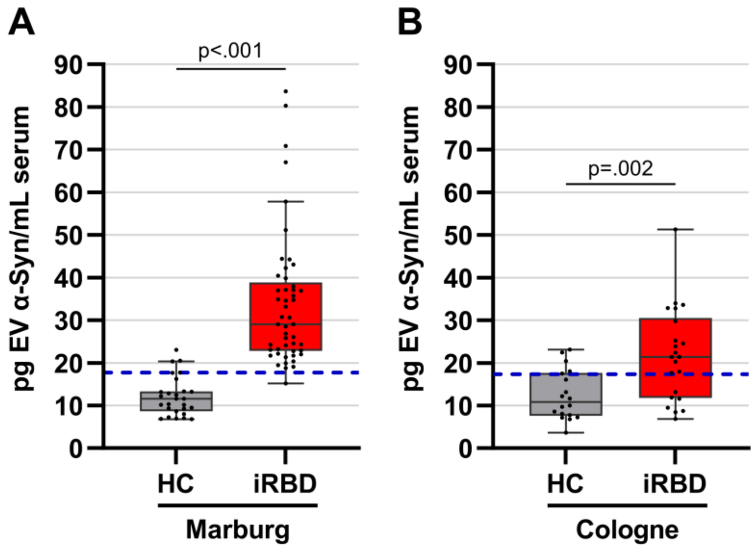


Boxplots showing the distribution of L1EV α-synuclein for **(A)** Marburg cohort **(B)** Cologne cohort. The threshold derived from Oxford Discovery (17.75 pg/mL) is shown with a dotted blue line. The midline of the box plots indicates the median, and the box indicates the 25th and 75th percentiles. Outliers and whiskers are plotted using the Tukey method. Statistical significance was determined by Mann-Whitney test.

© 2023 Yan et al. *JAMA Neurol.*

**eTable 2.** Summary of prodromal markers in validation group 2 (PPMI).

| No. (%) | | | |
|---|---|---|---|
| **Probability score** | **<5%** | **5-80%** | **>80%** |
| **Total No.** | 57 | 89 | 47 |
| **PSG-proven RBD** | 0 | 2 | 25 |
| **RBDSQ** | | | |
| Positive | 4 (7.0) | 22 (25.3) | 7 (31.8) |
| Negative | 53 (93.0) | 65 (74.7) | 15 (68.2) |
| Missing data | 0 | 0 | 0 |
| **DaT SPECT** | | | |
| Abnormal | 0 | 3 (3.4) | 30 (63.8) |
| Normal | 57 (100) | 83 (93.2) | 15 (31.9) |
| Missing data | 0 | 3 (3.4) | 2 (4.3) |
| **Subthreshold Parkinsonism** | | | |
| Positive | 0 | 6 (6.8) | 14 (29.8) |
| Negative | 57 (100) | 83 (93.2) | 33 (70.2) |
| Missing data | 0 | 0 | 0 |
| **Olfaction** | | | |
| Abnormal | 2 (3.5) | 24 (27.0) | 34 (72.3) |
| Normal | 55 (96.5) | 65 (73.0) | 11 (23.4) |
| Missing data | 0 | 0 | 2 (4.3) |
| **Constipation** | | | |
| Yes | 5 (8.8) | 27 (30.3) | 30 (63.8) |
| No | 52 (91.2) | 62 (69.7) | 17 (36.2) |
| Missing data | 0 | 0 | 0 |
| **EDS** | | | |
| Yes | 20 (35.1) | 58 (65.2) | 37 (78.7) |
| No | 37 (64.9) | 31 (34.8) | 10 (21.3) |
| Missing data | 0 | 0 | 0 |
| **Symptomatic OH** | | | |
| Yes | 2 (3.5) | 31 (34.8) | 23 (48.9) |
| No | 55 (96.5) | 58 (65.2) | 24 (51.1) |
| Missing data | 0 | 0 | 0 |
| **Erectile Dysfunction (in men)** | | | |
| Yes | 3 (4.8) | 17 (45.9) | 25 (71.4) |
| No | 18 (95.2) | 20 (54.1) | 10 (28.6) |
| Missing data | 0 | 0 | 0 |
| **Urinary Dysfunction** | | | |
| Yes | 13 (22.8) | 38 (42.7) | 29 (61.7) |
| No | 44 (77.2) | 51 (57.3) | 18 (38.3) |
| Missing data | 0 | 0 | 0 |
| **Depression (± anxiety)** | | | |
| Yes | 6 (10.5) | 17 (19.1) | 13 (27.7) |
| No | 51 (89.5) | 72 (80.9) | 34 (72.3) |
| Missing data | 0 | 0 | 0 |
| **Cognitive Deficit** | | | |
| Yes | 5 (8.8) | 22 (24.7) | 17 (36.2) |
| No | 52 (91.2) | 67 (75.3) | 30 (63.8) |
| Missing data | 0 | 0 | 0 |

Data shown as number of subjects, n and percentages, % in brackets. Percentages were calculated based on the total number shown for each group. Abbreviations: RBDSQ= REM Sleep Behaviour Disorder Screening Questionnaire; EDS= excessive daytime somnolence; OH= orthostatic hypotension.

© 2023 Yan et al. *JAMA Neurol.*

**eTable 3.** ROC analyses in derivation and validation groups.

| | AUC (95% CI) | Threshold (pg/mL) [a] | Sensitivity (95% CI) | Specificity (95% CI) |
|---|---|---|---|---|
| **Oxford Discovery** | | | | |
| iRBD vs HC | 0.85 (0.79-0.91) | 17.75 [b] | 0.77 (0.68-0.85) | 0.82 (0.72-0.89) |
| **Marburg+Cologne** | | | | |
| iRBD vs HC | 0.91 (0.86-0.96) | 18.41 | 0.86 (0.76-0.92) | 0.87 (0.74-0.94) |
| **PPMI** | | | | |
| PD vs <5% probability | 0.81 (0.72-0.89) | 16.02 | 0.79 (0.64-0.88) | 0.65 (0.51-0.77) |
| >80% vs <5% probability | 0.80 (0.71-0.89) | 20.35 | 0.72 (0.57-0.83) | 0.78 (0.64-0.87) |
| 5-80% vs <5% probability | 0.65 (0.56-0.75) | 13.19 | 0.71 (0.61-0.80) | 0.53 (0.39-0.66) |
| PD vs HC | 0.81 (0.70-0.92) | 21.00 | 0.61 (0.47-0.75) | 0.90 (0.70-0.98) |
| >80% vs HC | 0.80 (0.69-0.91) | 21.09 | 0.70(0.55-0.81) | 0.90 (0.70-0.98) |
| 5-80% probability vs HC | 0.65 (0.52-0.77) | 17.52 | 0.52 (0.41-0.62) | 0.80 (0.58-0.92) |
| >80% vs 5-80% probability | 0.67 (0.57-0.77) | 21.39 | 0.70 (0.55-0.81) | 0.60 (0.49-0.69) |

Abbreviations: iRBD= isolated REM Sleep Behaviour Disorder. AUC= area under the receiver operating characteristic curve.
[a] Optimal threshold determined by the Youden's index for each comparison and associated sensitivity and specificity are reported.
[b] This threshold was used for validation across cohorts as detailed in Figures 1 and 4.

© 2023 Yan et al. *JAMA Neurol.*

**eTable 4.** Difference in L1EV α-synuclein levels between *GBA1*$^{N409S}$ gene carriers with and without prodromal markers.

| *GBA1*$^{N409S}$ PM+ vs *GBA1*$^{N409S}$ PM- | | |
|---|---|---|
| | **Median difference (pg/mL)** | **p value** |
| **Prodromal marker** | | |
| Constipation | 6.18 | .01 |
| Subthreshold parkinsonism | 12.23 | .02 |
| Urinary Dysfunction | 5.34 | .02 |
| Cognitive deficit | 3.09 | .03 |
| RBDSQ | 1.98 | .06 |
| EDS | 1.77 | .06 |
| DaT SPECT | 9.50 | .07 |
| Depression (± anxiety) | 3.61 | .07 |
| Symptomatic OH | 0.81 | .09 |
| Erectile Dysfunction (in men) | 0.19 | .18 |
| Hyposmia | -2.04 | .89 |

Mann-Whitney test was used to compare L1EV α-Synuclein levels between two groups. Abbreviations: PM+=*GBA1*$^{N409S}$ with the indicated prodromal marker. PM-= *GBA1*$^{N409S}$ without any additional prodromal markers. RBDSQ= REM Sleep Behaviour Disorder Screening Questionnaire; EDS= excessive daytime somnolence; OH=orthostatic hypotension.

© 2023 Yan et al. *JAMA Neurol.*

**eFigure 5.** L1EV α-synuclein measurements in *GBA1*$^{N409S}$ gene carriers.

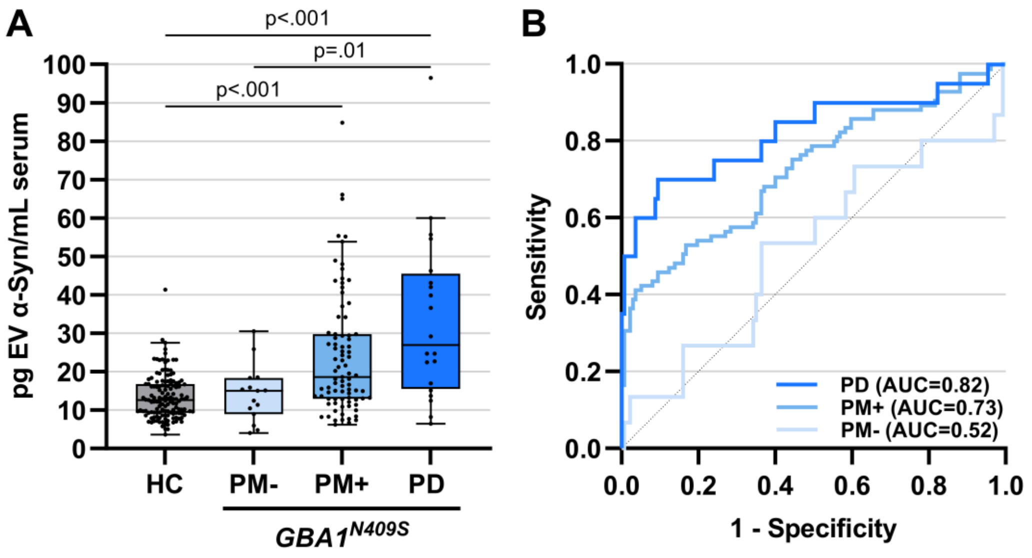


**(A)** Boxplot of L1EV α-synuclein measurements for HC, *GBA1*$^{N409S}$ non-manifest gene carriers without additional prodromal makers (PM-), *GBA1*$^{N409S}$ non-manifest gene carriers with any of the following markers (PM+): constipation or urinary dysfunction or subthreshold parkinsonism or cognitive deficit and *GBA1*$^{N409S}$ PD patients **(B)** ROC curves for the differentiation of *GBA1*$^{N409S}$ non-manifest gene carriers PM+ or PM- or *GBA1*$^{N409S}$ PD from healthy controls. Outliers and whiskers are plotted using the Tukey method. Statistical significance was determined by Kruskal-Wallis test.

© 2023 Yan et al. *JAMA Neurol.*

**eTable 5.** Association between L1EV α-synuclein and prodromal markers in iRBD.

| iRBD (n=127) | | | |
|---|---|---|---|
| Variable | β | SE | p value |
| Intercept | 1.288 | 0.2525 | <.001 |
| Age | $-1.70\times10^{-4}$ | 0.004 | .97 |
| Sex [Female] | -0.025 | 0.098 | .80 |
| Constipation [Yes] | -0.062 | 0.054 | .245 |
| Subthreshold parkinsonism [Positive] | 0.107 | 0.076 | .16 |
| Urinary dysfunction [Yes] | -0.033 | 0.054 | .55 |
| Cognitive deficit [Yes] | 0.116 | 0.052 | .03 |
| DaT SPECT [Abnormal] | -0.018 | 0.051 | .73 |
| Depression [Yes] | 0.039 | 0.058 | .50 |
| Symptomatic OH [Yes] | 0.050 | 0.058 | .39 |
| Olfaction [Abnormal] | 0.178 | 0.065 | .007 |
| Model R-square | .11 | | |
| Model p-value | .17 | | |

Multiple linear regression modelling identified hyposmia and cognitive dysfunction based on MoCA <26 as having the strongest association with L1EV α-synuclein. Abbreviations: iRBD= isolated REM Sleep Behaviour Disorder; OH= orthostatic hypotension.

© 2023 Yan et al. *JAMA Neurol.*

**eFigure 6.** L1EV α-synuclein in prodromal groups with available DaT SPECT.

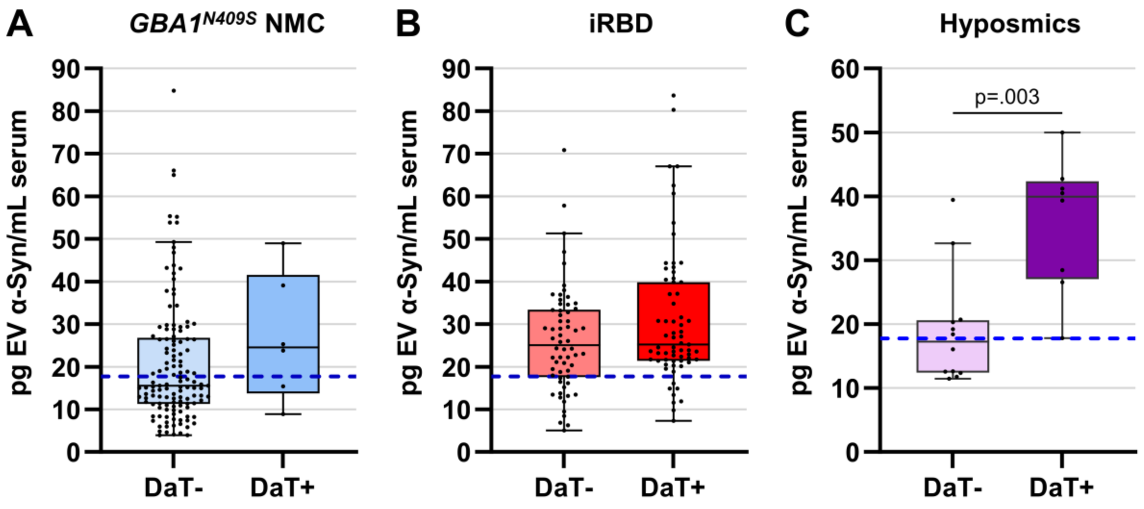


Comparison of those with negative (normal) and positive (abnormal) DaT SPECT across the individual prodromal groups of **(A)** *GBA1*$^{N409S}$ NMC, **(B)** iRBD and **(C)** Hyposmics. The threshold (17.75 pg/mL) derived from Oxford Discovery is shown with a dotted blue line. 42% of *GBA1*$^{N409S}$ NMC subjects, 74% of iRBD subjects and 50% of hyposmics with a normal DaT SPECT had L1EV α-synuclein levels above the trained threshold. The midline of the box plots indicates the median, and the box indicates the 25th and 75th percentiles. Outliers and whiskers are plotted using the Tukey method. Statistical significance was determined by Mann-Whitney test. NMC= non-manifest gene carriers

© 2023 Yan et al. *JAMA Neurol.*

**eFigure 7.** Correlation between L1EV α-synuclein and cognitive or motor scores.

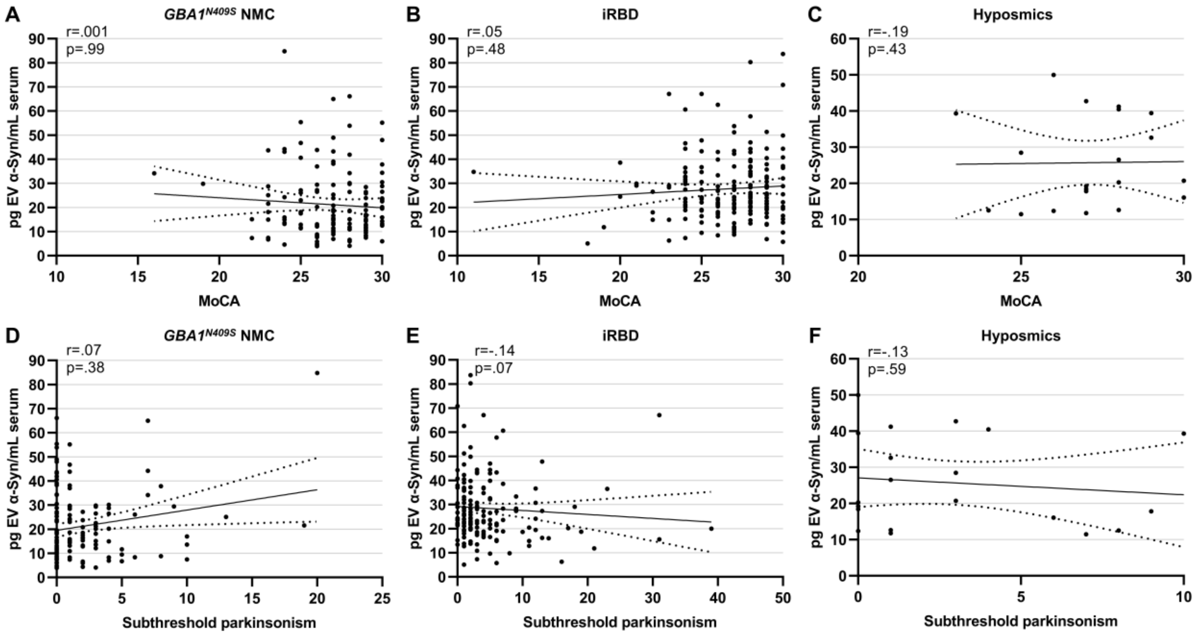


There was no correlation between MoCA or subthreshold parkinsonism as assessed by UPDRS III and L1EV α-synuclein levels in any of the three prodromal conditions. Least squares regression line with 95% CI was plotted and Spearman coefficient r with p value were reported for each group. NMC= non-manifest gene carriers.

© 2023 Yan et al. *JAMA Neurol.*

**eTable 6.** Correlation analyses in each cohort.

| Correlation coefficient r (p value) | | | | | | | |
|---|---|---|---|---|---|---|---|
| | | **HC** | **iRBD** | ***GBA1**^{N409S}* **NMC** | | | **Hyposmics** |
| **Oxford Discovery** | Age | .06 (.65) | .09 (.42) | .. | .. | .. | .. |
| | MoCA | .13 (.36) | -.01 (.91) | .. | .. | .. | .. |
| | Subthreshold Parkinsonism | -.08 (.58) | -.05 (.66) | .. | .. | .. | .. |
| **Marburg** | Age | .05 (.79) | -.07 (.63) | .. | .. | .. | .. |
| | MoCA | .74 (.13) | .03 (.84) | .. | .. | .. | .. |
| | Subthreshold Parkinsonism | -.12 (.84) | -.03 (.81) | .. | .. | .. | .. |
| **Cologne** | Age | .10 (.70) | .30 (.18) | | | | |
| | MoCA | .20 (.43) | .04 (.88) | .. | .. | .. | .. |
| | Subthreshold Parkinsonism | .. | .. | .. | .. | .. | .. |
| **PPMI** | Probability score | .. | .. | <5% | 5-80% | >80% | .. |
| | Age | -.28 (.23) | .14 (.52) | .01 (.93) | .04 (.72) | .56 (.05) | -.12 (.61) |
| | MoCA | -.19 (.41) | -.04 (.85) | .23 (.11) | .06 (.61) | -.07 (.83) | .19 (.43) |
| | Subthreshold Parkinsonism | -.003 (.99) | -.22 (.13) | .19 (.19) | -.13 (.26) | .32 (.28) | .13 (.59) |

Correlations between the indicated clinical parameters and L1EV $\alpha$-synuclein were calculated using Pearson correlation when data were normally distributed or Spearman correlation when data were not normally distributed. Correlation coefficient r with p value in brackets are shown for each parameter. NMC= non-manifest gene carriers.

**eTable 7.** Summary of prodromal markers in each cohort.

| No. (%) | | | | | | |
|---|---|---|---|---|---|---|
| **Cohort** | **Oxford** | **Marburg** | **Cologne** | **PPMI** | | |
| **Diagnosis** | **iRBD** | | | | ***GBA1*$^{N409S}$ NMC** | **Hyposmics** |
| **Total No.** | 97 | 51 | 24 | 27 | 146 | 20 |
| **PSG-proven RBD** | 97 (100) | 51 (100) | 24 (100) | 27 (100) | NA | NA |
| **RBDSQ** | | | | | | |
| Positive | NA | NA | NA | NA | 32 (13.0) | 0 |
| Negative | NA | NA | NA | NA | 114 (87.0) | 20 (100.0) |
| Missing data | NA | NA | NA | NA | 0 | 0 |
| **DaT SPECT** | | | | | | |
| Abnormal | 25 (25.8) | 23 (45.1) | 12 (50.0) | 19 (70.4) | 6 (4.1) | 8 (40.0) |
| Normal | 22 (22.7) | 27 (52.9) | 10 (41.7) | 8 (29.6) | 135 (92.5) | 12 (60.0) |
| Missing data | 50 (51.5) | 1 (2.0) | 2 (8.3) | 0 | 5 (3.4) | 0 |
| **Subthreshold Parkinsonism** | | | | | | |
| Positive | 27 (27.8) | 0 | 1 (4.2) | 4 (4.2) | 12 (8.2) | 4 (20.0) |
| Negative | 63 (65.0) | 50 (98.0) | 23 (95.8) | 23 (95.8) | 134 (91.8) | 16 (80.0) |
| Missing data | 7 (7.2) | 1 (2.0) | 0 | 0 | 0 | 0 |
| **Olfaction** | | | | | | |
| Abnormal | 61 (62.9) | 44 (86.3) | 21 (87.5) | 22 (81.5) | 19 (13.0) | 20 (100.0) |
| Normal | 25 (25.8) | 7 (13.7) | 3 (12.5) | 4 (14.8) | 126 (86.3) | 0 |
| Missing data | 11 (11.3) | 0 | 0 | 1 (3.7) | 0 | 0 |
| **Constipation** | | | | | | |
| Yes | 40 (41.2) | 17 (33.3) | 7 (29.2) | 14 (51.9) | 41 (28.1) | 7 (35.0) |
| No | 50 (51.6) | 32 (62.7) | 17 (70.8) | 13 (48.1) | 105 (71.9) | 13 (65.0) |
| Missing data | 7 (7.2) | 2 (2.0) | 0 (2.0) | 0 | 0 | |
| **EDS** | | | | | | |
| Yes | 56 (57.7) | 9 (17.6) | 5 (45.1) | 20 (74.1) | 81 (55.5) | 14 (70.0) |
| No | 33 (34.0) | 16 (31.3) | 19 (52.9) | 7 (25.9) | 65 (44.5) | 6 (30.0) |
| Missing data | 8 (8.3) | 26 (50.1) | 0 | 0 | 0 | 0 |
| **Symptomatic OH** | | | | | | |
| Yes | 13 (13.4) | 15 (29.4) | 9 (37.5) | 11 (40.7) | 38 (24.7) | 7 (35.0) |
| No | 74 (76.3) | 34 (66.7) | 10 (41.7) | 16 (59.3) | 108 (75.3) | 13 (65.0) |
| Missing data | 10 (10.3) | 2 (3.9) | 5 (20.8) | 0 | 0 | 0 |
| **Erectile Dysfunction (in men)** | | | | | | |
| Yes | 0 (23.0) | 21 (43.8) | 6 (28.6) | 15 (68.2) | 22 (26.0) | 9 (56.3) |
| No | 0 (24.7) | 23 (47.9) | 13 (61.9) | 7 (31.8) | 33 (74.0) | 7 (43.7) |
| Missing data | 92 (100) | 4 (8.3) | 2 (9.5) | 0 | 0 | 0 |
| **Urinary Dysfunction** | | | | | | |
| Yes | 51 (52.6) | 24 (47.1) | 10 (41.7) | 14 (51.9) | 53 (36.3) | 13 (65.0) |
| No | 39 (40.2) | 25 (49.0) | 14 (58.3) | 13 (48.1) | 93 (63.7) | 7 (35.0) |
| Missing data | 7 (7.2) | 2 (3.9) | 0 | 0 | 0 | 0 |
| **Depression (± anxiety)** | | | | | | |
| Yes | 27 (27.8) | 12 (23.5) | 9 (37.5) | 4 (14.8) | 27 (18.5) | 5 (25.0) |
| No | 63 (65.0) | 38 (74.5) | 15 (62.5) | 23 (85.2) | 119 (81.5) | 15 (75.0) |
| Missing data | 7 (7.2) | 1 (2.0) | 0 | 0 | 0 | 0 |
| **Cognitive Deficit** | | | | | | |
| Yes | 33 (34.0) | 9 (17.6) | 2 (8.3) | 7 (25.9) | 33 (22.6) | 4 (20.0) |
| No | 53 (54.7) | 40 (78.5) | 22 (91.7) | 20 (74.1) | 113 (77.4) | 16 (80.0) |
| Missing data | 11 (6.3) | 2 (3.9) | 0 | 0 | 0 | 0 |
| **Likelihood ratio** | | | | | | |
| Positive | 73 (75.3) | 48 (94.1) | 20 (83.3) | 25 (92.6) | 13 (8.9) | 9 (45.0) |
| Negative | 24 (24.7) | 3 (5.9) | 4 (16.7) | 2 (7.4) | 133 (91.1) | 11 (55.0) |

Data show number of subjects, n and percentage, % in brackets. Percentages were calculated based on the total number shown for each group. Abbreviations: iRBD= isolated REM Sleep Behaviour Disorder; NMC= non-manifest carriers; RBDSQ= REM Sleep Behaviour Disorder Screening Questionnaire; EDS= excessive daytime somnolence; OH= orthostatic hypotension; NA= non-applicable. Likelihood ratio was positive (>80% probability of having prodromal PD) or negative (<80% probability of having prodromal PD) for the indicated numbers and percentages across each cohort.

© 2023 Yan et al. *JAMA Neurol.*

**eFigure 8.** L1EV α-synuclein comparison between current and historic controls.

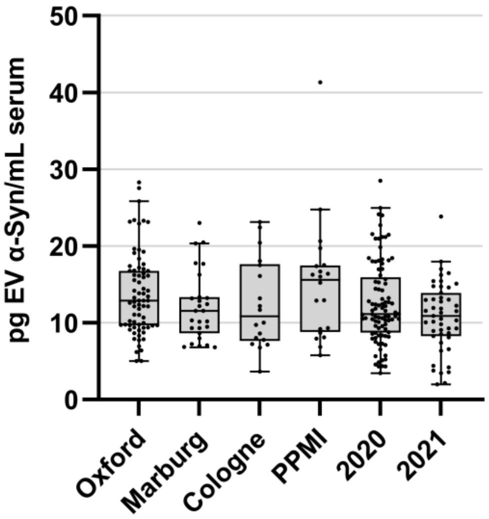


There was no difference between current healthy control samples analysed in this study (Oxford, Marburg, Cologne, PPMI) and samples from historic controls (2020 and 2021). The midline of the box plots indicates the median, and the box indicates the 25th and 75th percentiles. Outliers and whiskers are plotted using the Tukey method. Statistical significance was determined by Kruskal-Wallis test.

**eTable 8.** ROC analyses to stratify at-risk subjects.

| | AUC (95% CI) | Threshold (pg/mL) [a] | Sensitivity (95% CI) | Specificity (95% CI) |
|---|---|---|---|---|
| HC vs LR+ | 0.90 (0.87-0.93) | 18.54 | 0.81 (0.75-0.86) | 0.87 (0.83-0.91) |
| HC vs LR- | 0.70 (0.65-0.76) | 18.09 | 0.48 (0.41-0.56) | 0.86 (0.81-0.89) |
| LR+ vs LR- | 0.71 (0.65-0.76) | 18.65 | 0.81 (0.75-0.86) | 0.55 (0.48-0.63) |

Abbreviations: AUC= area under the receiver operating characteristic curve. LR= likelihood ratio.

[a] Optimal threshold determined by the Youden’s index for each comparison and associated sensitivity and specificity are reported.

© 2023 Yan et al. *JAMA Neurol.*

**eFigure 9.** L1EV-associated syntenin-1 levels in each condition.

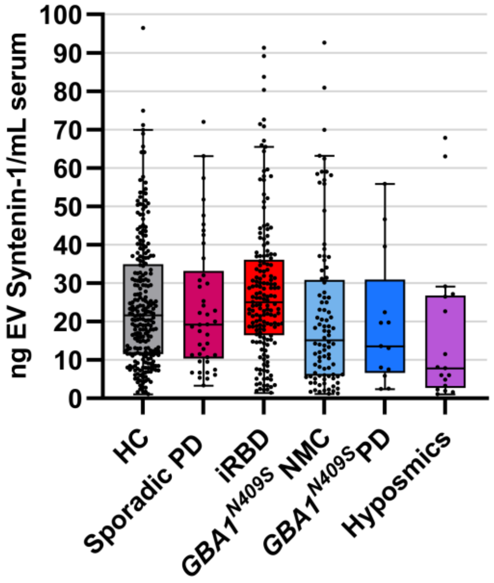


No specific pattern was observed for this generic EV marker across groups with different conditions. The midline of the box plots indicates the median, and the box indicates the 25th and 75th percentiles. Outliers and whiskers are plotted using the Tukey method. NMC= non-manifest gene carriers.

© 2023 Yan et al. *JAMA Neurol.*

**eFigure 10.** L1EV α-synuclein in prodromal groups with available SAA.

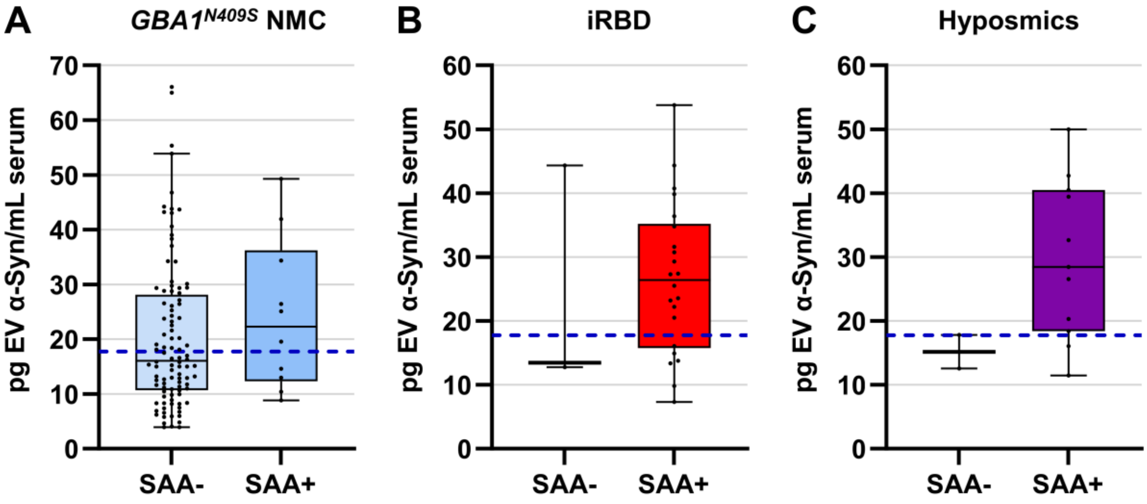


Comparison of those with negative (normal) and positive (abnormal) CSF SAA across the individual prodromal groups of **(A)** *GBA1*$^{N409S}$ non-manifest (NMC), **(B)** iRBD and **(C)** Hyposmics. The threshold derived from Oxford Discovery (17.75 pg/mL) is shown with a dotted blue line. The midline of the box plots indicates the median, and the box indicates the 25th and 75th percentiles. Outliers and whiskers are plotted using the Tukey method.

© 2023 Yan et al. *JAMA Neurol.*

**eFigure 11.** Correlation between serum L1EV α-synuclein and CSF total α-synuclein.

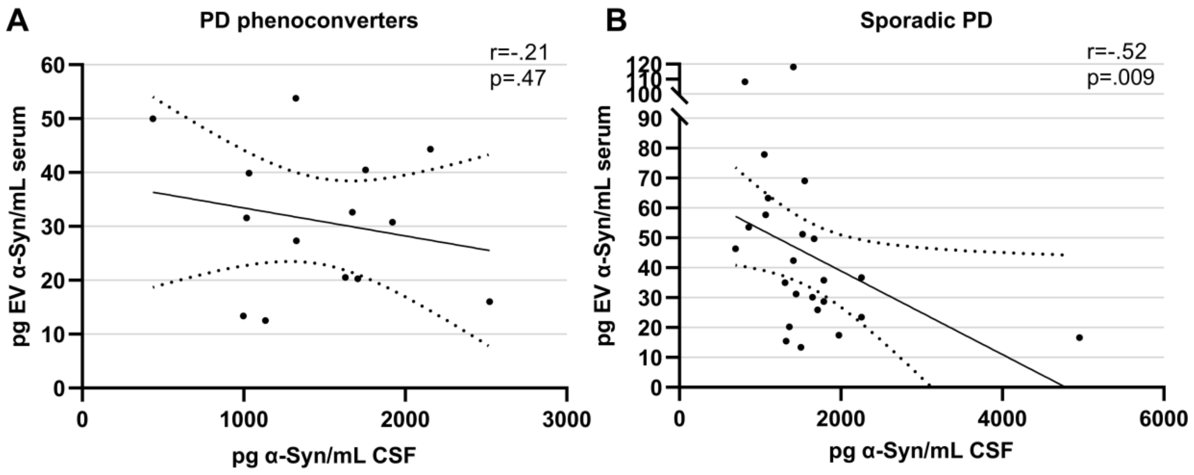


Inverse correlation between serum L1EV α-synuclein and CSF total α-synuclein levels was **(A)** weak in a small number of at-risk subjects who phenoconverted to PD and **(B)** significant in a larger group of patients with sporadic PD and available CSF measurements. Least squares regression line with 95% CI was plotted and Spearman coefficient r with p value were reported for each group.

© 2023 Yan et al. *JAMA Neurol.*

## eRefernces

1. Heinzel S, Berg D, Gasser T, et al. Update of the MDS research criteria for prodromal Parkinson's disease. *Mov Disord*. 2019;34(10):1464-1470. doi:10.1002/mds.27802
2. Janzen A, Vadasz D, Booij J, et al. Progressive Olfactory Impairment and Cardiac Sympathetic Denervation in REM Sleep Behavior Disorder. *J Parkinsons Dis*. 2022;12(6):1921-1935. doi:10.3233/JPD-223201
3. Jiang C, Hopfner F, Katsikoudi A, et al. Serum neuronal exosomes predict and differentiate Parkinson's disease from atypical parkinsonism. *J Neurol Neurosurg Psychiatry*. 2020;91(7):720-729. doi:10.1136/jnnp-2019-322588
4. Jiang C, Hopfner F, Berg D, et al. Validation of α-Synuclein in L1CAM-Immunocaptured Exosomes as a Biomarker for the Stratification of Parkinsonian Syndromes. *Mov Disord*. 2021;36(11):2663-2669. doi:10.1002/mds.28591
5. Graef. JD, Hoque. N, Polson. C, et al. Characterization of pathology-inducing α-synuclein species from human diseased brain tissue. *bioRxiv*. 2019. doi: 10.1101/588335v1
6. Youssef P, Kim WS, Halliday GM, Lewis SJG, Dzamko N. Comparison of Different Platform Immunoassays for the Measurement of Plasma Alpha-Synuclein in Parkinson's Disease Patients. *J Parkinsons Dis*. 2021;11(4):1761-1772. doi:10.3233/JPD-212694
7. Fu Y, Jiang C, Tofaris GK, Davis JJ. Facile Impedimetric Analysis of Neuronal Exosome Markers in Parkinson's Disease Diagnostics. *Anal Chem*. 2020;92(20):13647-13651. doi:10.1021/acs.analchem.0c03092

© 2023 Yan et al. *JAMA Neurol.*
