# Supplementary material for: Neuronally Derived Extracellular Vesicle α-Synuclein as a Serum Biomarker for Individuals at Risk of Developing Parkinson Disease
Source: JAMA Neurol. 2023 Dec 4;81(1):59–68. doi: 10.1001/jamaneurol.2023.4398 (PMC10696516; doi:10.1001/jamaneurol.2023.4398)
Supplement: Supplement 2. — Data sharing statement [file jamaneurol-e234398-s002.pdf]

Data Sharing Statement

Yan. Neuronally Derived Extracellular Vesicle α-Synuclein as a Serum Biomarker for Individuals at Risk of Developing Parkinson Disease. *JAMA Neurol*. Published December 04, 2023. doi:10.1001/jamaneurol.2023.4398

Data
Data available: Yes
Data types: Deidentified participant data
How to access data: Clinical data for the PPMI cohort should be requested via the PPMI portal (https://www.ppmi-info.org/).
When available: With publication

Supporting Documents
Document types: None

Additional Information
Who can access the data: Anonymized individual participant data and the study protocol will be shared with qualified parties on request to the corresponding author (George Tofaris).
Types of analyses: For biomarker or related studies
Mechanisms of data availability: By request to the corresponding author.
